# Aging-related immune phenotypes are associated with subclinical coronary atherosclerosis in a middle-aged population: a subcohort of the Swedish CArdioPulmonary bioImage Study

**DOI:** 10.1186/s12979-026-00600-9

**Published:** 2026-09-15

**Authors:** Rosanna W. S. Chung, Maike Schneider, Anna K. Lundberg, Lena Jonasson

**Affiliations:** 1 Department of Health, Medicine and Caring Sciences, Division of Diagnostics and Specialist Medicine, Linköping University, Linköping, Sweden; 2 Department of Cardiology, Linköping University Hospital, Linköping, 581 85 Sweden

## Abstract

Aging-related immune perturbations, including lower proportions of regulatory T (T_reg_) cells, shifts in naïve/memory T cells, and elevated monocyte and neutrophil counts, are well documented in patients with manifest coronary artery disease (CAD). In a cross-sectional study, we investigated whether these changes are present in subclinical coronary atherosclerosis.

A total of 1078 participants aged 50–64 (49% female) were consecutively recruited from the Swedish CArdioPulmonary bioImage Study (SCAPIS) cohort at Linköping University Hospital. Immune composition (25 subsets) was analyzed in fresh whole blood using flow cytometry. Atherosclerotic burden was assessed by coronary computed tomography angiography (CCTA) and coronary artery calcification (CAC) scoring. Subjects with a prior history of CAD (*n* = 11), active inflammation (*n* = 20), poor CCTA data (*n* = 19), missing CCTA data (*n* = 5), CT not performed (*n* = 5) or CCTA not performed (*n* = 51) were excluded.

Individuals with CCTA-detected coronary atherosclerosis (*n* = 327) showed higher levels of monocytes and central memory (CM) CD4^+^ T cells, and lower levels of naïve T_reg_ and naïve CD8^+^ T cells, compared to those without atherosclerosis (*n* = 640). Moreover, those with significant stenosis (≥50%, *n* = 40) had lower levels of naïve CD4^+^ T and CD56^bright^ NK cells. After adjustment for age and sex, increases in CM CD4^+^ T cells and reductions in effector memory (EM) CD4^+^ T, naïve T_reg_, and CD56^bright^ NK cells remained associated with ≥50% stenosis. After exploratory adjustment for other covariates and type 1 errors, changes in the proportions of CM and EM CD4^+^ T cells and naïve T_reg_ cells continued to show significant associations with subclinical atherosclerosis when subjects with ≥50% stenosis and those without were compared. No associations were observed between any immune cell subsets and CAC scores.

To conclude, immune perturbations, mainly related to an imbalance in naïve/memory CD4^+^ T cells and a reduction of naïve T_reg_ cells, are present already during the asymptomatic stage of CAD. The findings highlight the potential role of aging-related T cell phenotypes in the pathogenesis of atherosclerosis.

**Supplementary Information:**

The online version contains supplementary material available at https://doi.org/10.1186/s12979-026-00600-9.

## Introduction

Low-grade chronic inflammation in patients with cardiovascular disease is known to involve both the innate and adaptive immune system [1]. Analyses of human atherosclerotic lesions have consistently revealed abundant accumulations of monocyte-derived macrophages as well as CD4^+^ and CD8^+^ T cell subsets [2, 3]. In peripheral blood, elevated levels of innate immune cells, such as monocytes and granulocytes, have been shown to predict the future risk of cardiovascular disease [4, 5]. There is also considerable evidence of T cell activation, mainly involving CD4^+^ T cells, in the circulation of patients with manifest coronary artery disease (CAD), although mostly pronounced in those with acute coronary syndrome, i.e. unstable angina and myocardial infarction (MI) [6]. Inappropriately sustained T cell activation in atherosclerotic disease may drive T cell aging. Several features of T cell aging have been described in patients with manifest CAD. Such features include restricted repertoire of the T cell receptor [7, 8] and T cell senescence, defined by loss of CD28 and gain of NK cell markers CD56 and CD57 in predominantly the CD8^+^ T cell population [9, 10] Another hallmark of T cell aging, namely the gradual loss of naïve T cells and reciprocal increase of memory T cells in blood, has also been observed in patients with CAD [11]. In addition, previous studies have reported lower proportions of regulatory T (T_reg_) cells [12, 13] and, in particular naïve T_reg_ cells [14], in patients with CAD. T_reg_ cells are essential immunosuppressive regulators maintaining homeostasis of adaptive and innate immunity. Experimental evidence indicate that T_reg_ cells have an atheroprotective role. It has further been proposed that T_reg_ cell deficiency in blood may reflect a failure to regulate the adaptive and innate immunity [15, 16].

Whether T cell perturbations in blood predict the risk of future CAD in the population remains unclear. A recent study by Olson et al. [17], which used a nested case-cohort design, did not demonstrate any associations of circulating naïve and memory CD4^+^ T cell subsets or T_reg_ cells with the risk of coronary events. On the other hand, two other large prospective population-based cohort studies [18, 19] reported that reduced levels of T_reg_ cells were associated with increased risk of MI. Of note, those prospective studies used cryopreserved cells which is associated with major limitations, such as selective survival bias [20, 21].

A few previous population-based studies have investigated the cross-sectional relationship between peripheral T cell subsets and surrogate markers of subclinical atherosclerosis, with focus on ultrasound assessments of carotid arteries. Ammirati et al. [11] reported that effector memory (EM) T cells correlated with carotid intima-media thickness in a cohort of 118 individuals. Similarly, Olson et al. [22] found that memory CD4^+^ T cells were related to carotid intima-media thickness in the Multi-Ethnic Study of Atherosclerosis (MESA, *n* = 912). In line with this, an association between senescent T cell subsets and carotid arterial stiffness in the MESA cohort was recently reported [23].

So far, one previous study has investigated the associations between naïve and memory T cells and subclinical atherosclerosis in the coronary arteries. Olson et al. [22] showed that memory T cells were related to coronary artery calcium (CAC) scores in the MESA cohort. The use of CAC scores as a surrogate marker of coronary atherosclerosis may be effective for cardiovascular risk stratification. However, in contrast to coronary computed tomography angiography (CCTA), CAC does not distinguish between obstructive and non-obstructive CAD, neither does it provide information on the presence or burden of non-calcified plaques [24]. In a large random sample of the general population without established CAD, the Swedish CArdioPulmonary bioImage Study (SCAPIS, *n* = 25 182), 5.5% of the participants with CAC scores of 0 had any atherosclerosis as detected by CCTA while 7.0% of those with low CAC scores (up to 100) had coronary stenosis *≥* 50% [25].

Whether relationships can be found between immune cell compositions and subclinical coronary atherosclerosis assessed by CCTA has not been previously investigated. In the present study, our aim was to analyze the composition of immune cell subsets in fresh whole blood in a subcohort of SCAPIS and assess their relationships with subclinical atherosclerosis, measured by both CCTA and CAC scoring.

## Methods

### Study population

SCAPIS is a multicentre population-based prospective study comprising 30 000 individuals aged 50–64 recruited between 2013 and 2018 at six Swedish university hospitals [25]. Atherosclerotic coronary burden and CAC were assessed in all subjects by CCTA and non-contrast CT, respectively. The extent of CCTA-detected atherosclerosis was assessed in terms of luminal obstruction. “No atherosclerosis” indicated the absence of atherosclerosis, “Stenosis ≥ 50%” indicated more than or equal to 50% diameter luminal obstruction in any segment of the coronary artery tree, (i.e., significant stenosis). “Stenosis < 50%” indicated that only less than 50% diameter luminal obstruction in any segment of the coronary artery tree was detected [25]. The CAC categories (0, 1–10, 11–100, 101–400, and > 400) were based on a previous publication of the main SCAPIS cohort [25]. Data on anthropometry parameters, lifestyle, medication, prevalence of relevant diseases, family history of cardiovascular disease, office blood pressure and biochemical parameters were also provided by the SCAPIS cohort.

The current study analyzed data from a subcohort of SCAPIS (“SCAPIS Leukocyte”) which consisted of 1 078 participants. The subjects were consecutively recruited from the SCAPIS centre at Linköping University Hospital to establish an immune cell profile in whole fresh blood using flow cytometry [26, 27]. The subjects were excluded from the analysis due to the following reasons: CT not performed (*n* = 5); CCTA not performed (*n* = 51); poor quality of CCTA data (*n* = 19); previous MI or cardiac intervention (*n* = 11); active inflammation (C-reactive protein, CRP ≥ 10 mg/L) (*n* = 20); missing data in the CCTA data set (*n* = 5). The study used a cross-sectional design, which analyzed data collected at the baseline of the subcohort (Supplementary Fig. 1).

### Leukocyte subsets

The absolute counts of major immune cell types, including leukocytes, CD4^+^ and CD8^+^ T cells, B cells, granulocytes and monocytes, were performed on fresh whole blood using BD Multitest Trucount tubes (BD Biosciences, Sweden) as described [26]. The Trucount method was accredited by the Swedish Board for Accreditation and Conformity Assessment.

The composition of T cells and natural killer (NK) cell subsets was determined using multi-colour whole blood staining using the following monoclonal antibodies: anti-CD3 PerCP (Clone: SK7), anti-CD4 PE-Cy7 (Clone: SK3), anti-CD8 APC-H7 (Clone: SK1), anti-CD45RA FITC (Clone: L48), anti-CD25 APC (Clone: 2A3), anti-CD56 PE (Clone: NCAM16.2) and anti-CCR7 BV421 (Clone: 150,503). The staining procedure was previously described in detail [27]. The stained cells were analyzed on a FACS Canto II flow cytometer using FACSDiva software (BD Biosciences, Sweden). All reagents were purchased from BD Biosciences, Sweden. The definitions of cell subsets are listed in Supplementary Table 1. Subsets of T cells and NK cells were identified based on a gating strategy (Supplementary Fig. 2).

All flow cytometry analyses were performed at the Department of Clinical Immunology and Transfusion Medicine, Linköping University Hospital, Linköping, Sweden, where strict pre-analytical standardization was employed to maintain diagnostic accuracy. Sampling time was standardized to a narrow morning window. Instrument checks were performed daily against standardized controls. Inter- and intra-instrument stability were checked weekly to maintain a CV below 5%. Spectral compensation was performed with every batch of reagents as well as in regular time intervals. Gating reproducibility across analysts was evaluated by comparing the CVs of identical samples. All analysts were blinded to all subject information. No flow cytometry data were omitted due to poor data quality.

### Biochemical analyses

Biochemical parameters including total cholesterol, high-density lipoprotein (HDL) cholesterol, low-density lipoprotein (LDL) cholesterol, triglycerides and glycated hemoglobin (HbA1c) were determined in fasted plasma samples by the Department of Clinical Chemistry, Linköping University Hospital following manufacturers’ protocols. CRP was measured in plasma using an immunoturbidimetric assay with a Roche Cobas C502 analyzer (Roche Diagnostics, Scandinavia AB) with an inter-assay CV of 2.2%.

Interleukin (IL)-18 was quantified in plasma using the U-Plex Meso Scale Discovery (MSD) platform (Rockville, USA) following the manufacturer’s instructions. Analysis was performed at SciLifeLab Affinity Proteomics Unit, Uppsala, Sweden. Inter-assay CVs were 10.6%.

Cytomegalovirus (CMV) seropositivity, recognized as a major driving force behind T cell aging [28], was determined using immunochemical analysis for CMV IgG in serum based on standard clinical protocol. The analyses were performed by the Department of Clinical Microbiology, Linköping University Hospital, Sweden.

### Assessment of lifestyle and metabolic factors

Physical activity was measured using a tri-axial accelerometer (ActiGraph LCC, Pensacola, FL, USA) worn for seven days during conscious hours [29]. Sedentary activity was defined as 0-199 count per minute (cpm), and moderate and vigorous physical activity as ≥ 2690 cpm [30]. Alcohol intake was estimated using a validated food-frequency questionnaire Meal-Q (MiniMeal-Q) [31]. Body mass index (BMI) was calculated by dividing the body weight in kilograms by the square of height in meters. Abdominal obesity was defined as a waist-to-hip ratio of ≥ 0.85 for women and ≥ 0.90 for men.

### Statistical analysis

Statistical analyses were performed using IBM SPSS Statistics (version 31.0.1.0). Cohort characteristics across sex or atherosclerosis burden were compared using the Chi-square test for categorical variables and the Mann-Whitney U-test for continuous variables. Immune cell subset compositions across coronary stenosis categories or CAC score were compared using the Mann-Whitney U-test (comparisons between 2 groups) or the Kruskal-Wallis test (comparisons between all groups).

Multinomial logistic regression analysis was performed to assess the relationships between immune cell subsets and the extent of CCTA-detected atherosclerosis, or CAC score. All immune cell parameters were log_10_-transformed to meet assumptions of linearity. No zero values were present in the dataset. Possible confounders were identified using Spearman’s r correlations. Multinomial logistic regression analyses were performed in three models: Model 0 – unadjusted; Model 1 – adjusted for age and sex; and Model 2 – adjusted for age, sex, abdominal obesity, antihypertensive medication (self-reported), smoking status, diabetes mellitus (self-reported), plasma levels of HDL-cholesterol, plasma levels of IL-18 and CMV seropositivity. “No stenosis” and “CAC = 0” were used as the reference categories for the corresponding logistic regression analyses. Model 1 served as the primary core model as it preserves a stable events-per-variable ratio relative to the group with stenosis≥50% (*n* = 40). Model 2 serves as an additional exploratory full adjustment model where the cell subsets were further evaluated with additional adjustment for traditional cardiovascular risk factors. The data were presented as odds ratios and 95% confidence intervals. The odds ratios were obtained by transforming all exp(b) values using the following formula:

$$Odds\;Ratio_{10\%}=\mathrm{exp}{(b)}^{\log10(1.1)}$$

The obtained odds ratios represent the change in odds per 10% relative increase in the original (untransformed) cell count or proportion. Two-tailed *p*-values < 0.05 were considered significant.

Missing values were rare in this cohort. In total, 0.2% missing values were spread across 64 individuals in 7 out of 30 variables. Sensitivity analyses were conducted to test the robustness of our main findings. The strategies included modelling with various combinations of confounders in the multinomial logistic regression models, and conducting multiple imputation of missing values. No variable used in the multinomial regression models had more than 0.6% missing values. There was no change in direction or significance of the main findings. Subjects with missing values for a given variable were excluded from analyses involving that variable. To balance the risk of type 1 errors, the False Discovery Rate (*q*) was controlled to 25%, designated for exploratory immune profiling, using the Benjamini-Hochberg procedure [32] (Supplementary method).

## Results

### Cohort characteristics

The “SCAPIS Leukocyte” cohort (*n* = 972) resembled the characteristics of the national SCAPIS cohort [25]. Men showed a significantly higher prevalence of cardiovascular risk factors compared to women. A slightly higher proportion of women, compared to men, were CMV seropositive (*p* = 0.033) (Supplementary Table 2).

In terms of absolute counts of major cell types, the men had higher counts of leukocytes, granulocytes and monocytes, as well as higher granulocyte/lymphocyte ratios, while the women had higher proportions of B cells, NK cells, CD3^+^, and CD4^+^ T cells (Supplementary Table 2). As regards cell subset composition, the men had lower proportions of naïve CD4^+^ and CD8^+^ T cell and central memory (CM) CD8^+^ subsets, as well as higher proportions of CM and EM CD4^+^ and EM CD8^+^ T cell subsets. Furthermore, the men had higher proportions of both CD56^dim^ NK cells and CD8^+^ CD56^+^ T cells and lower proportions of CD56^bright^ NK cells, compared to women (Supplementary Table 2).

Similar to the national SCAPIS cohort [25], more men than women had CCTA-detected coronary atherosclerosis (45.0% vs. 21.9%, *p*<0.001) and CAC score > 0 (47.9% vs. 22.5%, *p*<0.001). Also, coronary stenosis ≥ 50% and calcified plaques regardless of stenosis severity (*p*<0.001 for both parameters) were more prevalent in men compared to women (Supplementary Table 3).

CCTA showed that 37 subjects had non-calcified plaques, amongst which 17 with non-calcified plaques only. In this latter group, 15 had CAC score = 0.

### Immune cell subset composition in relation to CCTA-detected atherosclerosis

To investigate the relationships between immune cell subsets and coronary atherosclerosis, subjects were stratified by the severity of coronary stenosis assessed by CCTA (Table 1). Male sex, increased age, abdominal obesity and use of antihypertensive and diabetic medication were more prevalent in those with any atherosclerosis compared to those without. Also, the subjects with any atherosclerosis showed higher blood pressure levels, lower levels of plasma HDL cholesterol and higher levels of plasma triglyceride, fasting glucose and IL-18, compared to those without atherosclerosis. There were no significant differences between subjects with stenosis ≥ 50% and those with stenosis < 50%, except for sex and CRP levels.

**Table 1:** Cohort characteristics stratified by the coronary atherosclerosis severity, assessed by coronary computed tomography angiography

	No atherosclerosis (*n*=640) A	Any atherosclerosis (*n*=327) B	Stenosis <50% (*n*=287) C	Stenosis ≥50% (*n*=40) D	*p*-value (A vs B)	*p*-value (A vs C)	*p*-value (A vs D)	*p*-value (C vs D)
Female sex, n (%)	372 (58.1)	105 (32.1)	99 (34.5)	6 (15.0)	**<0.001**	**<0.001**	**<0.001**	**0.013**
Age	55.6 (52.4–60.1)	59.2 (55.9–62.1)	59.1 (55.7–62.1)	59.9 (57.0-62.2)	**< 0.001**	**< 0.001**	**< 0.001**	0.290
Anthropometry								
BMI, kg m^− 2^	25.6 (23.4–28.8)	26.7 (24.2–29.9)	26.6 (24.2–29.9)	27.0 (24.7–29.7)	**0.001**	**0.003**	0.081	0.704
Abdominal obesity^a^, n (%)	367 (57.3)	253 (77.4)	220 (76.7)	33 (82.5)	**< 0.001**	**< 0.001**	**0.002**	0.408
Lifestyle								
Sedentary activity^b^, % of wear time	55 (47–61)	55 (49–62)	55 (49–61)	59 (49–64)	0.237	0.393	0.174	0.350
Physical activity^c^, % of wear time	6 (4–8)	6 (4–7)	6 (4–8)	6 (3–7)	0.205	0.361	0.144	0.289
Alcohol intake, g/day	5.5 (2.3–9.5)	6.7 (2.6–12.3)	6.7 (2.7–12.3)	6.5 (2.0-11.8)	**0.009**	**0.010**	0.381	0.890
Smoker current, n (%)	40 (6.3)	25 (7.6)	21 (7.3)	4 (10.0)	0.412	0.545	0.350	0.550
Smoker former, n (%)	171 (26.7)	113 (34.6)	98 (34.1)	15 (37.5)	**0.011**	**0.021**	0.138	0.676
Medication (self-reported)^d^								
Antihypertensive, n (%)	65 (10.2)	86 (26.3)	75 (26.1)	11 (27.5)	**< 0.001**	**< 0.001**	**< 0.001**	0.801
Cholesterol-lowering, n (%)	26 (4.1)	29 (8.9)	27 (9.4)	2 (5.0)	**0.002**	**0.001**	0.772	0.314
Diabetes medication, n (%)	14 (2.2)	27 (8.3)	23 (8.0)	4 (10.0)	**< 0.001**	**< 0.001**	**0.003**	0.474
Cancer medication, n (%)	2 (0.3)	2 (0.6)	2 (0.7)	0 (0.0)	0.493	0.409	0.723	0.596
Respiratory disease^e^ medication, n (%)	25 (3.9)	19 (5.8)	18 (6.3)	1 (2.5)	0.254	0.172	0.653	0.374
Rheumatic disease^f^ medication, n (%)	11 (1.7)	3 (0.9)	3 (1.0)	0 (0.0)	0.324	0.437	0.403	0.516
IBD^g^ medication, n (%)	11 (1.7)	5 (1.5)	4 (1.4)	1 (2.5)	0.827	0.717	0.716	0.593
Prevalence of relevant diseases (self-reported)								
Stroke, n (%)	2 (0.3)	3 (0.9)	3 (1.0)	0 (0.0)	0.107	0.060	0.882	0.607
Peripheral artery disease, n (%)	2 (0.3)	0 (0.0)	0 (0.0)	0 (0.0)	0.104	0.106	0.882	1.000
Rheumatic diseases^f^, n (%)	24 (3.8)	7 (2.1)	7 (2.4)	0 (0.0)	0.098	0.100	0.430	0.452
Sleep apnea, n (%)	15 (2.3)	16 (4.9)	14 (4.9)	2 (5.0)	**0.023**	**0.019**	0.546	0.754
Family history of cardiovascular diseases (self-reported)								
Family history of MI, n (%)	157 (24.5)	108 (33.0)	93 (32.4)	15 (37.5)	**0.010**	**0.020**	0.186	0.769
Family history of stroke, n (%)	161 (25.2)	89 (27.2)	80 (27.9)	9 (22.5)	0.143	0.057	0.597	0.261
Blood pressure^h^								
Systolic, mmHg	127 (116–141)	134 (124–144)	133 (123–143)	137 (127–151)	**< 0.001**	**< 0.001**	**< 0.001**	0.054
Diastolic, mmHg	81 (75–89)	84 (78–90)	83 (78–90)	85 (78–90)	**< 0.001**	**0.005**	**0.018**	0.244
Clinical chemistry								
Total cholesterol, mmol/L	5.4 (4.8–6.2)	5.5 (4.8–6.2)	5.5 (4.7–6.1)	5.7 (4.9–6.5)	0.922	0.819	0.294	0.241
LDL cholesterol, mmol/L	3.2 (2.6–3.8)	3.3 (2.7-4.0)	3.2 (2.7–3.9)	3.5 (3.0-4.3)	0.297	0.559	0.080	0.132
HDL cholesterol, mmol/L	1.6 (1.4-2.0)	1.5 (1.2–1.8)	1.5 (1.2–1.9)	1.3 (1.1–1.8)	**< 0.001**	**< 0.001**	**0.002**	0.109
Triglycerides, mmol/L	1.0 (0.7–1.4)	1.1 (0.8–1.6)	1.1 (0.8–1.6)	1.2 (0.8-2.0)	**0.003**	**0.011**	**0.027**	0.236
Fasted glucose, mmol/L	5.5 (5.2–5.9)	5.7 (5.4–6.2)	5.7 (5.4–6.1)	5.9 (5.5–6.6)	**< 0.001**	**< 0.001**	**< 0.001**	0.077
HbA1c, mmol/mol	35 (33–37)	35 (33–38)	35 (33–37)	36 (32–38)	**0.038**	0.055	0.281	0.818
CRP, mg/L	0.9 (0.5–1.8)	1.0 (0.5–1.9)	0.9 (0.5–1.8)	1.5 (0.6–2.3)	0.161	0.481	**0.010**	**0.022**
CMV seropositivity, n (%)	428 (66.9)	231 (70.6)	202 (70.4)	29 (72.5)	0.152	0.185	0.470	0.883
IL-18, pg/mL	325 (248–424)	359 (278–455)	357 (278–439)	375 (280–515)	**0.001**	**0.005**	**0.018**	0.214

The columns indicated were compared using the Mann-Whitney U (numerical data) or chi-squared-tests (categorical data). Data are given as median (interquartile range) or number (%). Statistically significant *p*-values were presented in bold

*BMI* Body mass index, *MI* Myocardial infarction, *IBD* Inflammatory bowel diseases, *LDL* Low-density lipoprotein, *HDL* High-density lipoprotein, *HbA1c* Hemoglobin A1c, *CRP* C-reactive protein, *CMV* Cytomegalovirus, *IL* Interleukin

^a^Abdominal obesity was defined as having a waist-to-hip ratio ≥ 0.85 (females) or ≥ 0.90 (males) according to the World Health Organization

^b^Sedative activity when wearing an accelerometer

^c^Moderate and vigorous physical activity when wearing an accelerometer

^d^Medication taken within 2 weeks of sampling

^e^Respiratory diseases include asthma, chronic obstructive pulmonary disease (COPD), chronic bronchitis, and emphysema

^f^Rheumatic diseases include rheumatoid arthritis, ankylosing spondylitis, psoriatic arthritis, systemic lupus erythematosus, and Sjögren’s syndrome

^g^Inflammatory bowel diseases include ulcerative colitis and Crohn’s disease

^h^Brachial blood pressure measured under hospital settings

**Fig. 1:**
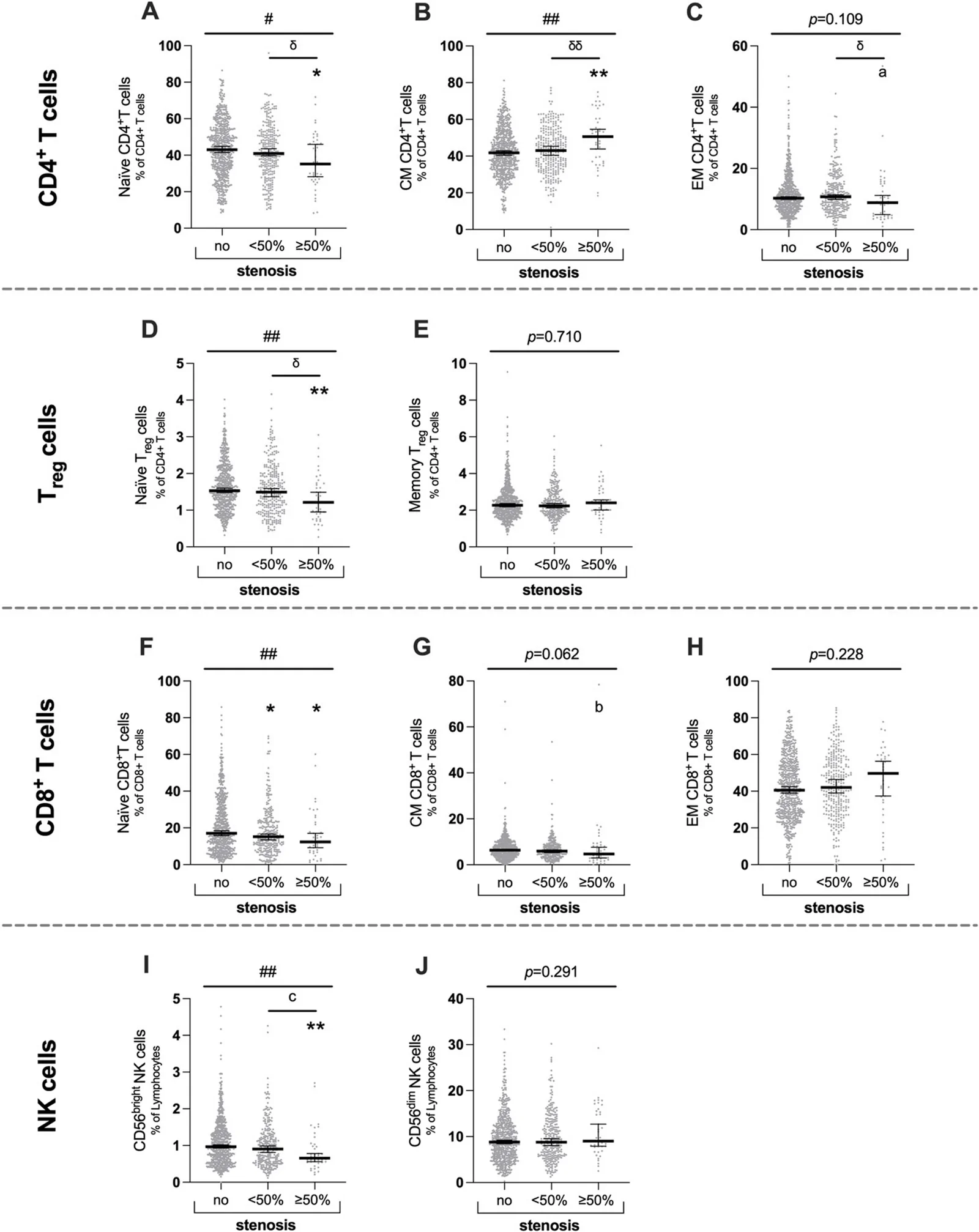
Circulating immune cell compositions stratified by coronary stenosis, assessed by coronary computed tomography angiography. The subjects were stratified by coronary atherosclerosis severity into “no stenosis”, “< 50% stenosis” or “*≥* 50% stenosis” groups. Immune cell composition in whole blood was analyzed using multi-color flow cytometry. Each dot represents a subject. **A-C**) CD4+ T cell subsets; **D-E**) Treg cell subsets; **F-H**) CD8+ T cell subsets; **I-J**) NK cell subsets. Multi-group comparisons were performed using the Kruskal-Wallis test. ^#^*p* < 0.05; ^##^*p* < 0.01; ^###^*p* < 0.001; Two-group comparisons were performed using the Mann-Whitney U-test. * *p* < 0.05, ** *p* < 0.01, *** *p* < 0.001, ^a^*p* = 0.055, ^b^*p* = 0.050 between subjects with stenosis vs. subjects without atherosclerosis; ^δ^*p* < 0.05, ^δδ^*p* < 0.01, ^c^*p* = 0.051 between subjects with stenosis < 50 vs. ≥ 50% stenosis. CM, central memory; EM, effector memory; NK, natural killer; T_reg_, regulatory CD4^+^ T. The “no stenosis” group includes subjects without any detectable coronary atherosclerosis

The absolute counts of leukocytes (*p* = 0.043) and monocytes (*p*<0.001) were higher in those with any atherosclerosis as compared to those without atherosclerosis (Supplementary Table 4). The difference in leukocyte counts became insignificant when subjects were stratified by atherosclerotic severity, i.e. stenosis < or *≥* 50% (Fig. 1A, Supplementary Table 5), while monocyte counts remained higher in those with stenosis below 50% (Fig. 1B, Supplementary Table 5). There were no differences between groups with and without atherosclerosis regarding granulocyte or lymphocyte counts, nor were there any differences in the proportions of B cells, NK cells, T cells, CD4^+^ or CD8^+^ T cell subsets (Supplementary Table 4).

The difference within the CD4^+^ T cell population became more obvious as the severity of coronary stenosis increased (Fig. 1, Supplementary Table 5). Subjects with coronary stenosis ≥ 50% had lower levels of naïve CD4^+^ T cells (*p* = 0.015, Fig. 1A), and naïve CD4^+^ T_reg_ cells (*p* = 0.001, Fig. 1D), as well as higher levels of CM CD4^+^ T cells (*p* = 0.001, Fig. 1A-B), compared to those without coronary atherosclerosis. On the other hand, the levels of EM CD4^+^ T cells only differed between those with < 50% stenosis and ≥ stenosis (Fig. 1C). As regards naïve CD8^+^ T cells, the levels were lower in both groups with atherosclerosis, i.e. stenosis < or *≥* 50%, compared to those without (Fig. 1F). None of the TEMRA CD4^+^ T cells, memory T_reg_ cells, the CM, EM or TEMRA CD8^+^ T cell subsets differed between groups (Fig. 1G-H, Supplementary Table 5).

There were also differences in the distribution of NK cell subsets between groups, as significantly lower proportions of CD56^bright^ NK cells were seen in those with stenosis ≥ 50% (*p* = 0.004, Fig. 1I), while CD56^dim^ NK cells did not differ (Fig. 1J). There were no differences in T cells expressing CD56^+^, neither within CD4^+^ nor CD8^+^ T cell populations (Supplementary Tables 4 and 5).

### Immune cell subset composition in relation to CAC scores

When the subjects were stratified into five categories of CAC scores (Supplementary Table 6), the differences in the composition of immune cell subsets were noticeably less pronounced compared to the stratification by CCTA-detected atherosclerosis. With increasing CAC scores, the leukocyte (*p* = 0.098) and monocyte counts increased (*p* = 0.001, Supplementary Fig. 3A-B), while the proportions of naïve CD8^+^ T cells decreased (*p* = 0.036, Supplementary Fig. 3C).

### Multivariate adjustment models of immune cell compositions in relation to coronary atherosclerosis

Before the relationships of immune cell parameters with either CCTA-detected atherosclerosis or CAC scores were explored, potential confounders were identified (Supplementary Fig. 4). All parameters that showed significant correlations with both atherosclerosis-related and cell-related parameters were included in the multivariate adjustment models. Age and sex were chosen for Model 1. Diabetes, abdominal obesity, smoking status, antihypertensive medication, HDL cholesterol, and IL-18 were added as confounders in subsequent multivariate adjustment analyses (Model 2). CMV seropositivity was included in Model 2 due to its strong relationship with naïve/memory T cell composition.

**Fig. 2:**
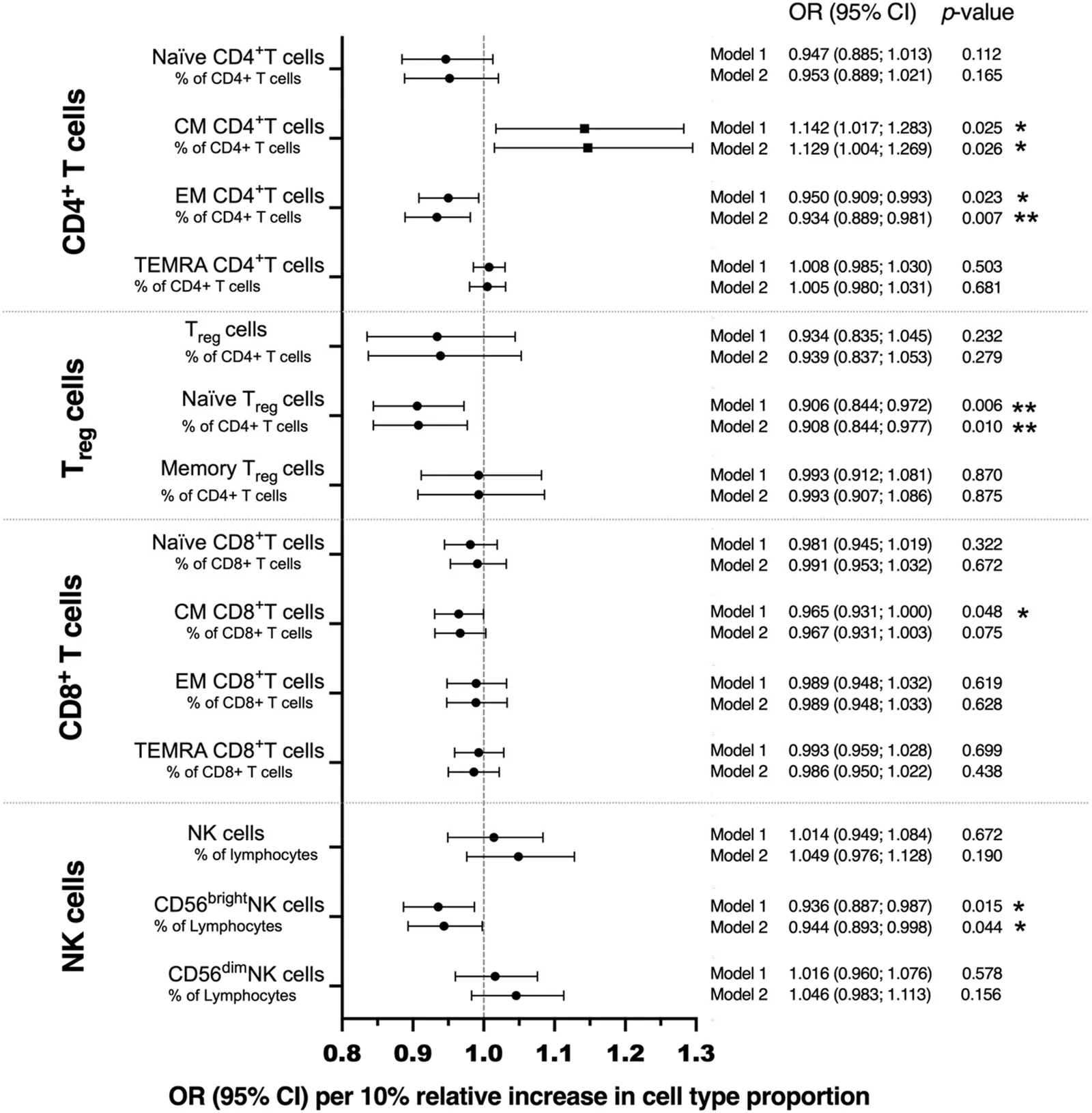
Multinomial logistic regression models depicting the relationship between immune cell composition and coronary atherosclerosis severity. The extent of coronary stenosis was assessed by coronary computed tomography angiography. Immune cell composition in whole blood was analyzed using multi-color flow cytometry. The odds ratios (OR) indicate the odds of having ≥ 50% atherosclerosis compared to no atherosclerosis for each 10% relative increase in the cell proportion. Model 1 was adjusted for age and sex (The primary core model). Model 2 was adjusted for age, sex, abdominal obesity, antihypertensive medication, smoking status, self-reported diabetes, plasma high-density lipoprotein-cholesterol, plasma interleukin-18, and CMV seropositivity (The exploratory model). All markers were log_10_-transformed before regression analyses. * *p* < 0.05; ** *p* < 0.01. CI, confidence interval. CM, central memory; EM, effector memory; TEMRA, terminally differentiated effector memory T cells re‑expressing CD45RA; T_reg_, regulatory T; NK, natural killer

The associations between immune cell composition and CCTA-detected atherosclerosis were explored in multinomial logistic regression models with multivariate adjustment (Fig. 2, Supplementary Tables 7–8). Neither the counts of leukocytes, monocytes, granulocytes, lymphocytes, nor the proportions of B cells, NK cells, T cells, CD4^+^ and CD8^+^ T cell subsets showed any significant associations with CCTA-detected atherosclerosis (Supplementary Table 7).

Among the subset of CD4^+^ T cells (Supplementary Table 8), the proportions of naïve CD4^+^ T, EM CD4^+^ T, and naïve T_reg_ cells were negatively associated, while CM CD4^+^ T cells were positively associated with coronary stenosis ≥ 50% before adjustment. After full adjustment, EM CD4^+^ T cells (OR = 0.934, *p* = 0.007), CM CD4^+^ T cells (OR = 1.148, *p* = 0.026) and naïve T_reg_ cells (OR = 0.909, *p* = 0.011) remained associated with coronary stenosis ≥ 50% (Fig. 2, Supplementary Table 8).

Within the CD8^+^ T cell population, the proportions of naïve CD8^+^ and CM CD8^+^ T cells showed a negative association with coronary stenosis ≥ 50% before adjustment. These associations did not remain significant after full adjustment (Fig. 2, Supplementary Table 7).

Regarding NK cell subsets, the proportions of CD56^bright^ NK cells (OR = 0.944, *p* = 0.043) remained negatively associated with coronary stenosis ≥ 50% after full adjustment (Fig. 2, Supplementary Table 8).

The results were adjusted using the Benjamini-Hochberg procedure to minimize type 1 error. Almost all cell types that were initially significant remained significant in Models 1 and 2, except CD56^bright^ NK cells in Model 2 when subjects with no stenosis were compared to subjects with stenosis ≥ 50% (Supplementary Table 9).

No significant associations were found between any cell subsets and coronary stenosis < 50% after adjustments in Model 2 (Supplementary Tables 7–8). As the majority of our subjects had either only calcified plaques or a mixed of calcified and noncalcified plaques, it was not possible to determine any difference in cell compositions between subjects with only calcified plaques and those with only noncalcified plaques.

Furthermore, the associations between immune cell parameters and CAC scores were investigated. Before adjustment, leukocyte and monocyte counts showed significant associations with higher CAC scores (Supplementary Table 10). The associations became non-significant after adjustment for age and sex.

## Discussion

Our study is the first to demonstrate a relationship between aging-related changes in the adaptive immune system and subclinical coronary atherosclerotic burden, assessed by CCTA. Naïve T cell subsets, including naïve CD4^+^ T cells and naïve CD8^+^ T cells, were reduced as coronary stenosis severity increased from < 50% to ≥ 50% stenosis. Conversely, the CM CD4^+^ T cells showed a stepwise increase with coronary stenosis severity. Collectively, the findings are in line with the notion that T cell aging plays a role in early atherosclerosis, not only reflecting the later clinically manifest stage of disease.

A naïve/memory T cell imbalance in circulating blood, defined by a reduction in naïve T cells and an accumulation of memory T cells, is a hallmark of T cell aging [33]. Such an imbalance has been reported in patients with stable CAD [11, 14] as well as in patients with acute coronary syndrome [34]. However, previous population-based studies, which have investigated the associations between naïve/memory CD4^+^ T cell imbalance and subclinical atherosclerosis, have mainly relied on surrogate markers, such as carotid intima-media thickness. For example, Olson et al. [22] demonstrated that a decrease in naïve CD4^+^ T cells, along with an increase in memory CD4^+^ T cells, was associated with carotid intima-media thickness in the MESA cohort. On the other hand, Ammirati et al. [11] showed a positive association between EM CD4^+^ T cells and carotid intima-media thickness in healthy subjects, but no associations with naïve or CM CD4^+^ T cells. This is in contrast to our findings, which showed that CCTA-detected atherosclerosis was positively associated with CM CD4^+^ T cells, but negatively associated with EM CD4^+^ T cells. The CM CD4^+^ T cells and EM CD4^+^ T cells are traditionally believed to differentiate in a stepwise manner from naïve CD4^+^ T cells. However, according to a recent review by Künzli and Masopust [35], the differentiation of these subsets is not strictly hierarchical. It may be assumed that an increased fraction of CM CD4^+^ T cells, along with a decreased fraction of naïve CD4^+^ T cells, reflects a shift towards immune aging [36]. It is also worth noting that the majority of memory T cells does not reside in circulating blood. In the context of atherosclerosis, an accumulation of memory T cells within the atherosclerotic plaque has been proposed to exacerbate local inflammation and plaque instability [37]. Interestingly, a recent single-cell RNA sequencing study showed high proportions of CM CD4^+^ T cells in culprit lesions from patients with acute coronary syndrome, and further suggested that a differentiation from CM CD4^+^ T cells to effector T cells might occur in the plaque [38].

Interestingly, the proportion of naïve T_reg_ cells was independently and negatively associated with the presence of coronary stenosis ≥ 50% in all adjustment models. T_reg_ cell deficiency has been consistently demonstrated in patients with manifest CAD compared to healthy control subjects [12–14] but whether a similar deficiency occurs already in subclinical atherosclerosis has been largely unknown. However, a recent study including 77 healthy elderly men demonstrated that the presence of subclinical carotid atherosclerosis, defined by ultrasound, was associated with a lower percentage of naïve T_reg_ cells [39]. Furthermore, higher T_reg_ cell levels have been associated with lower CAC score progression in the MESA cohort [40]. It is well known that T_reg_ cells exert regulatory effects in both innate and adaptive immunity [41]. Furthermore, an increasing body of experimental evidence indicates an atheroprotective role [42]. The naïve T_reg_ cell pool serves as a reservoir for the immunoregulatory effector T_reg_ cells [41]. Thus, besides supporting a state of immune aging, the present findings allow us to speculate that the immunoregulatory and atheroprotective capacity is diminished in individuals with subclinical coronary atherosclerosis.

The significantly increased plasma levels of CRP and IL-18 in subjects with coronary stenosis ≥ 50% compared to those without atherosclerosis further support a state of accelerated immune aging in the former group, since both CRP and IL-18 have been consistently associated with inflammatory-mediated aging [43–45]. Also, the percentages of CD56^bright^ NK cells in lymphocytes were significantly lower in subjects with coronary stenosis ≥ 50%. NK cells represent a critical innate immune population whose role in atherosclerosis is not fully elucidated, largely due to contrasting evidence [46]. Still, the findings are in line with premature immune aging, as aging has been associated with a decline in peripheral CD56^bright^ NK cells [47, 48].

CD56^+^ T cells, or T cells expressing NK cell-associated receptors such as CD56 and CD57, represent another cell population that has been associated with aging [49]. Also, peripheral CD56^+^ T cells have been shown to be expanded in patients with manifest CAD compared to healthy control subjects [10, 50]. However, we found no relationship between CD56^+^ T cell subsets and subclinical coronary atherosclerosis in the present cohort.

Of note, there were no correlations between any immune cell subset and coronary atherosclerosis when we used CAC scores as a surrogate measure. The results may reflect the well-known limitations of CAC scoring, such as the inability to detect non-calcified as well as obstructive plaques [51]. In the MESA cohort, Patel et al. [40] showed that increased levels of memory CD4^+^ T cells were related to CAC scores. However, when comparing their study to our present work, one might note that Patel et al. used cryopreserved cells which are associated with methodological challenges, such as poor viability and potential selection of cell subsets [20, 21]. Also, the subjects in the current SCAPIS cohort may have a generally lower atherosclerosis burden with a median CAC score of 38 compared to 72 in the MESA cohort.

In a few previous population-based studies, circulating monocyte and granulocyte counts, as well as granulocyte-lymphocyte ratios, have been associated with carotid atherosclerosis and CAC scores [4, 52, 53]. In the present study, we found no significant associations between any of these parameters and coronary atherosclerosis. However, it is worth noting that monocytes and granulocytes are highly heterogeneous cell populations. Associations between specified subsets and early atherosclerosis may occur, e.g., non-classical monocytes have been shown to correlate with carotid intima-media thickness progression [54].

Aging-associated shifts in immune composition, such as reduction of naïve CD4^+^ T cells and naïve T_reg_ cells and elevation of CM CD4^+^ cells, showed several correlations with traditional cardiovascular risk factors, in particular male sex, but also diabetes, abdominal obesity, hypertension and low HDL cholesterol levels. The results are in line with previous studies which have shown that male sex as well as obesity is associated with accelerated immune aging [55, 56]. However, along with increased signs of immune aging, men also had more traditional cardiovascular risk factors and a larger burden of coronary atherosclerosis than women. Still, one intriguing possibility is that immune aging has a major impact on the well-documented sex differences in the development and severity of atherosclerosis [25].

There are several limitations in our study. First, the study population may be considered relatively healthy with the majority (66%) showing no coronary atherosclerosis. Also, a majority (88%) of the subjects with CCTA-detected atherosclerosis was in a relatively early stage of disease manifestation (< 50% stenosis). The group with stenosis ≥ 50% (*n* = 40) provided limited power; therefore, the findings should be considered exploratory. Also, the effect of sex on cell composition in those with ≥ 50% stenosis was unable to be investigated (female *n* = 6). Although the number of subjects who may require immunosuppressive or immunomodulatory treatments was minimal, the relevant information was not available to conduct further exclusion. Note that the memory T_reg_ subset, in the absence of FOXP3 or CD127 in the current phenotypic definition, may be slightly overestimated due to potential contamination of activated helper T cells. The conclusion regarding naïve T_reg_ was not affected. Finally, perturbations in immune cell activities were not delineated.

In conclusion, by combining analyses of immune cell composition in fresh whole blood with CCTA quantification of atherosclerotic plaque burden, we demonstrate that age-associated immune cell perturbations, including an imbalance in naïve/memory CD4^+^ T cells and a reduction of naïve T_reg_ cells, are present in subjects with subclinical coronary atherosclerosis, particularly in those with ≥ 50% stenosis. Although no causal effects can be deduced from this cross-sectional study, it is intriguing to speculate that strategies targeting aging-related immune changes might halter the silent progression of subclinical atherosclerosis.

## Supplementary Information

Supplementary Material 1: Supplementary Fig. 1. Flow chart of exclusion and inclusion procedures. Supplementary Fig. 2. The gating strategy for the subsets of CD4+ and CD8+ T cells and NK cells in whole blood. Supplementary Fig. 3. Circulating immune cell compositions stratified by coronary artery calcium (CAC) score categories. Supplementary Fig. 4. A heatmap indicating the Spearman correlation coefficients between atherosclerosis-related parameters, immune cell compositions and potential confounders. Supplementary Table 1. Definitions of leukocyte subsets used in the gating strategies of output from flow cytometry. Supplementary Table 2. Cohort characteristics stratified by sex. Supplementary Table 3. Prevalence of coronary atherosclerosis, assessed by coronary computed tomography angiography or coronary artery calcium scoring, stratified by sex. Supplementary Table 4. Immune cell composition in subjects stratified by the presence of coronary atherosclerosis, assessed by coronary computed tomography angiography. Supplementary Table 5. Immune cell composition in subjects stratified by the extent of coronary atherosclerosis, assessed by coronary computed tomography angiography. Supplementary Table 6. Immune cell composition in subjects stratified by coronary artery calcium score categories. Supplementary Table 7. Logistic regression models between the major immune cell types and atherosclerosis burden, assessed by coronary computed tomography angiography (*n* = 967). Supplementary Table 8. Logistic regression models between immune cell composition and coronary atherosclerosis burden, assessed by coronary computed tomography angiography (*n* = 967). Supplementary Table 9. Adjustment of results using the Benjamini-Hochberg procedure. Supplementary Table 10. Logistic regression models between immune cell composition and coronary artery calcium score (CAC) (*n *= 967).

## Data Availability

The SCAPIS Leukocyte study is part of a large nationwide cross-sectional cohort called SCAPIS. The majority of the data describing subject characteristics were provided by the SCAPIS Data Access Board and are not available for public sharing due to confidentiality regulations. If the request is reasonable, data may be shared, provided that permissions are also obtained from the Swedish Ethical Review Authority and the SCAPIS Data Access Board.
