# Supplementary material for: Aging-related immune phenotypes are associated with subclinical coronary atherosclerosis in a middle-aged population: a subcohort of the Swedish CArdioPulmonary bioImage Study
Source: Immun Ageing. 2026 Sep 15;23:33. doi: 10.1186/s12979-026-00600-9 (PMC13576287; doi:10.1186/s12979-026-00600-9)
Supplement: Supplementary file 1 [file 12979_2026_600_MOESM1_ESM.docx]

**Supplementary Method**

**Benjamini-Hochberg procedure**

The Benjamini-Hochberg (BH) procedure was carried out by listing all *p*-values, corresponding to all immune cell parameters generated from the multinomial logistic regression analyses. The *p*-values were then sorted in ascending order and assigned rank (*i*) where the smallest *p*-value ranked 1*.* The BH critical value was calculated using the following formula, where the number of models (*m*) = 26; False Discovery Rate (*q*) = 0.25.

$$BH critical value= \frac{i}{m} q$$

A threshold was determined by comparing the *p*-values and the BH critical values in descending order of rank. The highest-ranked *p*-value that is less than or equal to its BH critical value is the threshold. Any lower-ranked *p*-value from the threshold and the threshold were considered significant.

**Supplementary Figure 1**. **Flow chart of exclusion and inclusion procedures.** CT, computed tomography; CCTA, Coronary CT angiography; MI, myocardial infarction; PCI, Percutaneous coronary intervention; CABG, Coronary artery bypass grafting; CRP, C-reactive protein.

**Supplementary Figure 2**. **The gating strategy for the subsets of CD4^+^ and CD8^+^ T cells and NK cells in whole blood.** A) Viable lymphocytes were gated based on size (i.e., forward scatter (FSC)) and granularity (i.e., side scatter (SSC)). B) From the “lymphocyte” gate, natural killer (NK) and CD56^+^ T cells were identified based on CD3 and CD56 expression. Subsequently, CD56^bright^ NK and CD56^dim^ NK cells were identified by the levels of CD56. C) From the “Lymphocyte” gate, CD3^+^ T cells were identified based on SSC and CD3 expression. D) From the “CD3^+^ “gate, CD4^+^ and CD8^+^ T cells were gated based on CD4 and CD8 expression, respectively. From the “CD3^+^CD56^+^ gate, CD4^+^CD56^+^ T cells and CD8^+^CD56^+^ T cells were identified based on CD4 and CD8 expression. E) From the “CD4^+^” and “CD8^+^” gates, naïve, central memory (CM), effector memory (EM) and terminally differentiated effector memory T cells re‑expressing CD45RA (TEMRA) T cell subsets were gated and defined as stated in Supplementary Table 1. F) From the “CD4^+^“ gate, naïve and memory T_reg_ cells were identified as CD25^++^CD45RA^+^ and CD25^++^CD45RA^-^, respectively. G) The naïve and memory Treg cells identified in F) were backgated by displaying gate-specific coloring on the CD45RA^+^ and CD45RA^-^ populations in CD25 and SSC plots. T_reg_ cells were defined as CD25^++^ with relatively lower granularity compared to the rest of the T cells in the respective plots.

**Supplementary Figure 3**. **Circulating immune cell compositions stratified by coronary artery calcium (CAC) score categories.** Each dot represents a subject. Multi-group comparisons were performed using the Kruskal-Wallis test. # *p* < 0.05; ## *p* < 0.01; Two-group comparisons were performed using the Mann-Whitney U-test. * *p* < 0.05; ** *p* < 0.01; *** *p* < 0.001 for CAC>0 vs CAC=0; CAC, coronary artery calcium; ns, not significant.

**Supplementary Figure 4**. **A heatmap indicating the Spearman correlation coefficients between atherosclerosis-related parameters, immune cell compositions and potential confounders.** Atherosclerosis was assessed by coronary computed tomography angiography or coronary calcium scores (5 categories). Factors that showed no significant correlations were excluded from this figure. All cell parameters were log_10_-transformed. The color gradient reflects the strength and direction of correlations, with blue indicating negative correlations and purple indicating positive correlations. CAC, coronary artery calcium; CM, central memory; EM, effector memory; TEMRA, terminally differentiated effector memory T cells re‑expressing CD45RA; T_reg_, regulatory CD4^+^ T; NK, natural killer, BMI, body mass index; HDL, high-density lipoprotein, LDL, low-density lipoprotein; CMV, Cytomegalovirus; CRP, C-reactive protein; IL, interleukin. * *p* < 0.05; ** *p* < 0.01; *** *p* < 0.001.

**Supplementary Table 1**. **Definitions of leukocyte subsets used in the gating strategies of output from flow cytometry**

| **Full names** | **Cell types** | **Definitions** |
| --- | --- | --- |
|  | B cells | CD19^+^ |
|  | T cells | CD3^+^ |
|  | CD4^+^ T cells | CD3^+^CD4^+^ |
|  | Naïve CD4^+^ T cells | CD3^+^CD4^+^CD45RA^+^CCR7^+^ |
| Central memory CD4^+^ T cells | CM CD4^+^ T cells | CD3^+^CD4^+^CD45RA^-^CCR7^+^ |
| Effector memory CD4^+^ T cells | EM CD4^+^ T cells | CD3^+^CD4^+^CD45RA^-^CCR7^-^ |
| Terminally differentiated effector memory T cells re‑expressing CD45RA CD4^+^ T cells | TEMRA CD4^+^ T cells | CD3^+^CD4^+^CD45RA^+^CCR7^-^ |
| Regulatory CD4^+^ T cells | T_reg_ cells | Sum of Naive T_reg_ cells and memory T_reg_ cells |
|  | Naïve T_reg_ cells | CD3^+^CD4^+^CD25^++^CD45RA^+^ |
|  | Memory T_reg_ cells^1^ | CD3^+^CD4^+^CD25^++^CD45RA^-^ |
|  | CD8^+^ T cells | CD3^+^CD8^+^ |
|  | Naïve CD8^+^ T cells | CD3^+^CD8^+^CD45RA^+^CCR7^+^ |
| Central memory CD8^+^ T cells | CM CD8^+^ T cells | CD3^+^CD8^+^CD45RA^-^CCR7^+^ |
| Effector memory CD4^+^ T cells | EM CD8^+^ T cells | CD3^+^CD8^+^CD45RA^-^CCR7^-^ |
| Terminally differentiated effector memory T cells re‑expressing CD45RA CD8^+^ T cells | TEMRA CD8^+^ T cells | CD3^+^CD8^+^CD45RA^+^CCR7^-^ |
| Natural killer cells | NK cells | CD3^-^CD56^+^ |
|  | CD56^+^ T cells | CD3^+^CD56^+^ |
|  | CD4^+^ CD56^+^ T cells | CD3^+^CD4^+^CD56^+^ |
|  | CD8^+^ CD56^+^ T cells | CD3^+^CD8^+^CD56^+^ |

^1^ Potential contamination of activated helper T cells.

**Supplementary Table 2**. **Cohort characteristics stratified by sex.**

|  | **Total (n=967)** |  | **Women (n=477)** | **Men (n=490)** | *p*-value |
| --- | --- | --- | --- | --- | --- |
| Female sex, n (%) | 477 (49.3) |  | - | - | - |
| Age | 57.1 (53.2-60.9) |  | 56.3 (52.9-60.7) | 57.9 (53.9-61.1) | **0.014** |
| **Anthropometry** | | | | | |
| BMI, kg m^-2^ | 26.1 (23.7-29.0) |  | 25.1 (22.6-29.1) | 26.7 (24.7-29.0) | **<0.001** |
| Abdominal obesity^1^, n (%) | 620 (64.1) |  | 213 (44.7) | 407 (83.1) | **<0.001** |
| **Lifestyle** | | | | | |
| Sedentary activity^2^, % of wear time | 55 (48-61) |  | 53 (46-59) | 57 (50-63) | **<0.001** |
| Physical activity^3^, % of wear time | 6 (4-8) |  | 6 (4-8) | 6 (4-8) | 0.705 |
| Alcohol intake, g/day | 6.0 (2.4-10.4) |  | 4.1 (1.8-7.3) | 8.4 (3.9-13.9) | **<0.001** |
| Smoker current, n (%) | 65 (6.7) |  | 30 (6.3) | 35 (7.1) | 0.596 |
| Smoker former, n (%) | 284 (29.4) |  | 149 (31.2) | 135 (27.6) | 0.208 |
| **Medication (self-reported)^4^** | | | | | |
| Antihypertensive, n (%) | 151 (15.6) |  | 58 (12.2) | 93 (19.0) | **0.003** |
| Cholesterol-lowering, n (%) | 55 (5.7) |  | 19 (4.0) | 36 (7.3) | **0.024** |
| Diabetes, n (%) | 41 (4.2) |  | 17 (3.6) | 24 (4.9) | 0.303 |
| Cancer medication, n (%) | 4 (0.4) |  | 3 (0.6) | 1 (0.2) | 0.303 |
| Respiratory disease^5^ medication, n (%) | 44 (4.6) |  | 18 (3.8) | 26 (5.3) | 0.316 |
| Rheumatic diseases^6^ medication, n (%) | 14 (1.4) |  | 8 (1.7) | 6 (1.2) | 0.556 |
| IBD^7^ medication, n (%) | 16 (1.7) |  | 7 (1.5) | 9 (1.8) | 0.653 |
| **Prevalence of relevant diseases (self-reported)** | | | | | |
| Stroke, n (%) | 5 (0.5) |  | 3 (0.6) | 2 (0.4) | 0.656 |
| Peripheral artery disease, n (%) | 2 (0.2) |  | 2 (0.4) | 0 (0.0) | 0.263 |
| Sleep apnea, n (%) | 31 (3.2) |  | 8 (1.7) | 23 (4.7) | **0.021** |
| **Family history of cardiovascular disease (self-reported)** | | | | | |
| Family history of MI, n (%) | 265 (27.4) |  | 152 (31.9) | 113 (23.1) | **0.004** |
| Family history of stroke, n (%) | 250 (25.9) |  | 137 (28.7) | 113 (23.1) | 0.106 |
| **Blood pressure^8^** | | | | | |
| Systolic, mmHg | 129 (119-142) |  | 127 (116-140) | 132 (122-144) | **<0.001** |
| Diastolic, mmHg | 82 (76-89) |  | 81 (75-88) | 83 (76-90) | **0.042** |
| **Clinical chemistry** | | | | | |
| Total cholesterol, mmol/L | 5.4 (4.8-6.2) |  | 5.6 (5.0-6.3) | 5.3 (4.6-6.0) | **<0.001** |
| LDL cholesterol, mmol/L | 3.2 (2.6-3.9) |  | 3.2 (2.6-3.9) | 3.2 (2.6-3.9) | 0.805 |
| HDL cholesterol, mmol/L | 1.6 (1.3-2.0) |  | 1.8 (1.5-2.2) | 1.4 (1.2-1.7) | **<0.001** |
| Triglycerides, mmol/L | 1.0 (0.8-1.4) |  | 0.9 (0.7-1.3) | 1.2 (0.8-1.7) | **<0.001** |
| Fasting glucose, mmol/L | 5.6 (5.3-6.0) |  | 5.5 (5.1-5.8) | 5.7 (5.4-6.1) | **<0.001** |
| HbA1c, mmol/mol | 35 (33-37) |  | 35 (33-37) | 35 (32-37) | 0.515 |
| CRP, mg/L | 0.9 (0.5-1.9) |  | 0.9 (0.5-1.9) | 0.9 (0.5-1.8) | 0.857 |
| CMV seropositivity, n (%) | 659 (68.1) |  | 341 (71.5) | 318 (64.9) | **0.038** |
| IL-18, pg/mL | 339 (256-432) |  | 297 (235-381) | 381 (299-482) | **<0.001** |
| **Immune cell counts** | | | | | |
| *Major cell types* | | | | | |
| Leukocytes, $\text{×10}^{\text{6}}\text{cells/L}$ | 5690 (4714-6659) |  | 5534 (4591-6530) | 5834 (4841-6846) | **0.002** |
| Lymphocytes $\text{×10}^{\text{6}}\text{cells/L}$ | 1765 (1463-2105) |  | 1776 (1489-2113) | 1762 (1435-2103) | 0.671 |
| Granulocytes, $\text{×10}^{\text{6}}\text{cells/L }$ | 3220 (2570-3990) |  | 3106 (2456-3906) | 3358 (2728-4104) | **<0.001** |
| Granulocyte/lymphocyte ratio | 1.83 (1.42-2.31) |  | 1.78 (1.33-2.22) | 1.87 (1.53-2.38) | **<0.001** |
| Monocytes, $\text{×10}^{\text{6}}\text{cells/L}$ | 370 (301-461) |  | 337 (283-416) | 407 (329-497) | **<0.001** |
| B cells, % of lymphocytes | 11 (8-14) |  | 11 (8-14) | 10 (8-14) | **0.043** |
| NK cells, % of lymphocytes | 12 (8-16) |  | 11 (7-14) | 9 (13-17) | **<0.001** |
| T cells, % of lymphocytes | 77 (72-81) |  | 78 (73-81) | 76 (70-80) | **<0.001** |
| CD4^+^ T cells, % of lymphocytes | 50 (44-56) |  | 52 (45-57) | 48 (42-54) | **<0.001** |
| CD8^+^ T cells, % of lymphocytes | 24 (19-31) |  | 23 (19-30) | 25 (19-31) | 0.196 |
| *Cell subsets* | | | | | |
| Naïve CD4^+^ T cells, % of CD4^+^ T cells | 42 (32-53) |  | 45 (34-55) | 40 (29-50) | **<0.001** |
| CM CD4^+^ T cells, % of CD4^+^ T cells | 42 (34-52) |  | 41 (33-50) | 44 (35-53) | **0.002** |
| EM CD4^+^ T cells, % of CD4^+^ T cells | 10 (7-15) |  | 10 (7-13) | 11 (8-16) | **0.006** |
| TEMRA CD4^+^ T cells, % of CD4^+^ T cells | 1 (0-2) |  | 1 (0-2) | 1 (0-2) | 0.466 |
| T_reg_ cells, % of CD4^+^ T cells | 3.87 (3.23-4.67) |  | 3.87 (3.23-4.60) | 3.87 (3.24-4.80) | 0.696 |
| Naïve T_reg_ cells, % of CD4^+^ T cells | 1.51 (1.11-1.99) |  | 1.53 (1.13-2.03) | 1.50 (1.08-1.96) | 0.231 |
| Memory T_reg_ cells, % of CD4^+^ T cells | 2.27 (1.82-2.82) |  | 2.22 (1.84-2.72) | 2.33 (1.80-2.98) | 0.331 |
| Naïve CD8^+^ T cells, % of CD8^+^ T cells | 16 (9-28) |  | 20 (12-32) | 14 (8-23) | **<0.001** |
| CM CD8^+^ T cells, % of CD8^+^ T cells | 6 (4-9) |  | 7 (4-9) | 6 (3-9) | **0.001** |
| EM CD8^+^ T cells, % of CD8^+^ T cells | 41 (29-55) |  | 40 (29-52) | 43 (29-57) | **0.037** |
| TEMRA CD8^+^ T cells, % of CD8^+^ T cells | 25 (13-41) |  | 23 (13-38) | 27 (14-45) | **0.008** |
| CD56^bright^ NK cells, % of lymphocytes | 0.94 (0.58-1.34) |  | 0.99 (0.61-1.37) | 0.88 (0.55-1.31) | **0.015** |
| CD56^dim^ NK cells, % of lymphocytes | 9 (6-12) |  | 8 (5-11) | 10 (7-14) | **<0.001** |
| CD56^+^ T cells, % of T cells | 8 (4-13) |  | 7 (4-12) | 9 (5-14) | **<0.001** |
| CD4^+^ CD56^+^ T cells, % of CD4^+^ T cells | 1 (0-2) |  | 1 (0-2) | 1 (0-3) | 0.390 |
| CD8^+^ CD56^+^ T cells, % of CD8^+^ T cells | 17 (10-26) |  | 15 (9-25) | 18 (12-27) | **<0.001** |
|  |  |  |  |  |  |

Men and women were compared using the Mann-Whitney U (numerical data) or chi-squared-tests (categorical data). Data are given as median (interquartile range) or number (%). Statistically significant *p*-values were presented in bold.
^1^ Abdominal obesity was defined as having a waist-to-hip ratio ≥0.85 (females) or ≥0.90 (males) according to the World Health Organization.
^2^ Sedative physical activity when wearing an accelerometer.
^3^ Moderate and vigorous physical activity when wearing an accelerometer.

^4^ Medication taken within 2 weeks of sampling.

^5^ Respiratory diseases include asthma, chronic obstructive pulmonary disease (COPD), chronic bronchitis, and emphysema.

^6^ Rheumatic diseases include rheumatoid arthritis, ankylosing spondylitis, psoriatic arthritis, systemic lupus erythematosus, and Sjögren’s syndrome.

^7^ IBD include ulcerative colitis and Crohn’s disease.

^8^ Brachial blood pressure measured in a hospital setting.

BMI, body mass index; IBD, inflammatory bowel disease; MI, myocardial infarction; LDL, low-density lipoprotein; HDL, high-density lipoprotein; HbA1c, hemoglobin A1c; CRP, C-reactive protein; CMV, cytomegalovirus; IL, interleukin; NK, natural killer; CM, central memory; EM, effector memory; TEMRA, terminally differentiated effector memory T cells re‑expressing CD45RA; T_reg_, regulatory CD4^+^ T.

**Supplementary Table 3.** **Prevalence of coronary atherosclerosis, assessed by coronary computed tomography angiography or coronary artery calcium scoring, stratified by sex.**

|  | **Total (n=967)** |  | **Women (n=477)** | **Men (n=490)** | *p*-value |
| --- | --- | --- | --- | --- | --- |
| **Coronary atherosclerosis burden** | | | | | |
| No atherosclerosis | 640 (66.2) |  | 372 (78.0) | 268 (54.7) | **<0.001** |
| Any form of atherosclerosis | 327 (33.8) |  | 105 (22.0) | 222 (45.3) | **<0.001** |
| Any stenosis <50% | 287 (29.7) |  | 99 (20.8) | 188 (38.4) | **<0.001** |
| Any stenosis ≥50% | 40 (4.1) |  | 6 (1.3) | 34 (6.9) | **<0.001** |
| One-vessel disease | 30 (3.1) |  | 5 (1.0) | 25 (5.1) | **<0.001** |
| Two-vessel disease | 6 (0.6) |  | 1 (0.2) | 5 (1.0) | 0.108 |
| Three-vessel disease | 0 (0.0) |  | 0 (0.0) | 0 (0.0) | - |
| Left main disease | 1 (0.1) |  | 0 (0.0) | 1 (0.2) | 0.324 |
| Proximal LAD disease | 13 (1.3) |  | 3 (0.6) | 10 (2.0) | 0.057 |
| Left main, proximal LAD or three-vessel  disease | 14 (1.4) |  | 3 (0.6) | 11 (2.2) | **0.035** |
| Any calcified plaque | 310 (32.1) |  | 96 (20.1) | 214 (43.7) | **<0.001** |
| Any calcified stenosis ≥ 50% | 30 (3.1) |  | 4 (0.8) | 26 (5.3) | **<0.001** |
| Any noncalcified plaque | 37 (3.8) |  | 13 (2.7) | 24 (4.9) | 0.078 |
| Any noncalcified stenosis ≥ 50% | 12 (1.2) |  | 3 (0.6) | 9 (1.8) | 0.090 |
| Only calcified plaques | 290 (30.0) |  | 92 (19.3) | 198 (40.4) | **<0.001** |
| Only noncalcified plaques | 17 (1.8) |  | 9 (1.9) | 8 (1.6) | 0.764 |
| **Coronary artery calcium score** | | | | | |
| = 0 | 624 (64.5) |  | 369 (77.4) | 255 (52.0) | **<0.001** |
| > 0 | 343 (35.5) |  | 108 (22.6) | 235 (48.0) | **<0.001** |
| 1 to 10 (ultralow) | 85 (8.8) |  | 36 (7.5) | 49 (10.0) | 0.178 |
| 11 to 100 (low) | 153 (15.8) |  | 53 (11.1) | 100 (20.4) | **<0.001** |
| 101 to 400 (moderate) | 74 (7.7) |  | 16 (3.4) | 58 (11.8) | **<0.001** |
| > 400 (high) | 27 (2.8) |  | 3 (0.6) | 24 (4.9) | **<0.001** |
| Median CAC^1^ | 38 (10-134) |  | 22 (7-68) | 49 (12-182) | **<0.001** |

Men and women were compared using the Mann-Whitney U (numerical data) or chi-squared-tests (categorical data). Data are given as median (interquartile range) or number (%). Statistically significant *p*-values were presented in bold.
^1^ Subjects with CAC score = 0 were excluded.

LAD, Left anterior descending; CAC, coronary artery calcium.

**Supplementary Table 4**. **Immune cell composition in subjects stratified by the presence of coronary atherosclerosis, assessed by coronary computed tomography angiography**.

|  | **No atherosclerosis (n=640)** | | **Any atherosclerosis (n=327)** | | *p*-value |
| --- | --- | --- | --- | --- | --- |
| *Major cell types* | | | | | |
| Leukocytes $\text{ ×10}^{\text{6}}\text{cells/L }$ | 5612 (4673-6610) | | 5775 (4826-6824) | | **0.043** |
| Lymphocytes $\text{ ×10}^{\text{6}}\text{cells/L}$ | 1752 (1427-2070) | | 1786 (1515-2171) | | 0.062 |
| Granulocytes $\text{ ×10}^{\text{6}}\text{cells/L }$ | 3212 (2549-3970) | | 3230 (2617-4067) | | 0.106 |
| Granulocyte/  Lymphocyte ratio | 1.84 (1.39-2.32) | | 1.78 (1.48-2.28) | | 0.998 |
| Monocytes $\text{ ×10}^{\text{6}}\text{cells/L}$ | 360 (290-448) | | 394 (322-490) | | **<0.001** |
| B cells,  % of lymphocytes | 11 (8-14) | | 10 (8-13) | | 0.269 |
| NK cells,  % of lymphocytes | 12 (8-15) | | 11 (8-16) | | 0.857 |
| T cells,  % of lymphocytes | 76 (72-81) | | 77 (72-81) | | 0.362 |
| CD4^+^ T cells,  % of lymphocytes | 50 (44-56) | | 50 (44-56) | | 0.506 |
| CD8^+^ T cells,  % of lymphocytes | 24 (19-31) | | 23 (19-30) | | 0.506 |
| *Cell subsets* | | | | | |
| **CD4^+^ T cell subsets, % of CD4^+^ T cells** | | | |  | |
| Naïve CD4^+^ T cells | 43 (32-54) | | 41 (31-51) | | 0.097 |
| CM CD4^+^ T cells | 42 (33-50) | | 44 (35-53) | | **0.049** |
| EM CD4^+^ T cells | 10 (7-14) | | 11 (7-15) | | 0.992 |
| TEMRA CD4^+^ T cells | 1 (0-2) | | 1 (0-2) | | 0.613 |
| **T_reg_ cell subsets, % of CD4^+^ T cells** | | |  | |  |
| T_reg_ cells | 3.90 (3.26-4.74) | | 3.80 (3.16-4.62) | | 0.123 |
| Naïve T_reg_ cells | 1.53 (1.15-2.06) | | 1.46 (0.99-1.95) | | **0.019** |
| Memory T_reg_ cells | 2.27 (1.82-2.81) | | 2.25 (1.84-2.87) | | 0.806 |
| **CD8^+^ T cell subsets,** **% of CD8^+^ T cells** | | | |  |  |
| Naïve CD8^+^ T cells | 17 (9-30) | | 15 (9-24) | | **0.005** |
| CM CD8^+^ T cells | 6 (4-9) | | 6 (4-8) | | 0.050 |
| EM CD8^+^ T cells | 41 (28-54) | | 43 (30-57) | | 0.126 |
| TEMRA CD8^+^ T cells | 24 (13-41) | | 27 (14-41) | | 0.226 |
| **NK cells, % of lymphocytes** | | | |  |  |
| CD56^bright^ NK cells | 0.97 (0.61-1.39) | | 0.91 (0.56-1.28) | | 0.058 |
| CD56^dim^ NK cells | 9 (6-12) | | 9 (6-13) | | 0.607 |
| **CD56^+^ T cells subsets** | |  |  | |  |
| CD56^+^ T cells,  % of T cells | 8 (4-13) | | 8 (4-13) | | 0.977 |
| CD4^+^ CD56^+^ T cells,  % CD4^+^ T cells | 1 (0-2) | | 1 (0-3) | | 0.314 |
| CD8^+^ CD56^+^ T cells,  % CD8^+^ T cells | 17 (10-25) | | 17 (11-26) | | 0.750 |

Groups were compared using the Mann-Whitney U test. Data is given as median (interquartile range). Data for the major cell types were generated from absolute counts of each immune cell type. Data for the cell subsets were generated from relative counts using multi-color flow cytometry. Statistically significant *p*-values were presented in bold.
NK, natural killer; CM, central memory; EM, effector memory; TEMRA, terminally differentiated effector memory T cells re‑expressing CD45RA; T_reg_, regulatory CD4^+^ T.

**Supplementary Table 5**. **Immune cell composition in subjects stratified by the extent of coronary atherosclerosis, assessed by coronary computed tomography angiography**.

|  | | **A**  **No stenosis (n=640)** | | **B**  **Stenosis < 50%**  **(n=287)** | | | **C**  **Stenosis ≥ 50% (n=40)** | *p*-value (between all columns) | *p*-value (between A and B) | *p*-value (between A and C) | *p*-value (between  B and C) |
| --- | --- | --- | --- | --- | --- | --- | --- | --- | --- | --- | --- |
|  | *Major cell types* | | | | | | | | | |  |
| Leukocytes $\text{ ×10}^{\text{6}}\text{cells/L }$ | | 5612 (4673-6610) | | 5747 (4842-6773) | | | 5927 (4531-6842) | 0.116 | 0.071 | 0.210 | 0.623 |
| Lymphocytes $\text{ ×10}^{\text{6}}\text{cells/L}$ | | 1752 (1427-2070) | | 1771 (1497-2171) | | | 1859 (1542-2236) | 0.132 | 0.115 | 0.154 | 0.391 |
| Granulocytes $\text{ ×10}^{\text{6}}\text{cells/L }$ | | 3212 (2549-3970) | | 3208 (2602-4061) | | | 3408 (2682-4221) | 0.238 | 0.163 | 0.255 | 0.611 |
| Granulocyte/  Lymphocyte ratio | | 1.84 (1.39-2.32) | | 1.78 (1.48-2.28) | | | 2.01 (1.46-2.35) | 0.628 | 0.998 | 0.358 | 0.318 |
| Monocytes $\text{ ×10}^{\text{6}}\text{cells/L}$ | | 360 (290-448) | | 396 (322-494) | | | 381 (325-440) | **<0.001** | **<0.001** | 0.209 | 0.627 |
| B cells,  % of lymphocytes | | 11 (8-14) | | 10 (8-13) | | | 10 (7-14) | 0.519 | 0.325 | 0.491 | 0.695 |
| NK cells,  % of lymphocytes | | 12 (8-15) | | 11 (8-16) | | | 12 (9-19) | 0.334 | 0.857 | 0.160 | 0.228 |
| T cells,  % of lymphocytes | | 76 (72-81) | | 77 (72-81) | | | 76 (68-80) | 0.342 | 0.362 | 0.305 | 0.180 |
| CD4^+^ T cells,  % of lymphocytes | | 50 (44-56) | | 50 (44-56) | | | 50 (43-56) | 0.763 | 0.506 | 0.826 | 0.623 |
| CD8^+^ T cells,  % of lymphocytes | | 24 (19-31) | | 23 (19-30) | | | 25 (15-30) | 0.573 | 0.506 | 0.380 | 0.505 |
|  | *Cell subsets* | | | | | | | | | |  |
| **CD4^+^ T cell subsets, % of CD4^+^ T cells** | | | | |  |  | | | | |  |
| Naïve CD4^+^ T cells | | 43 (32-54) | | 41 (33-51) | | | 35 (27-48) | **0.038** | 0.302 | **0.015** | **0.047** |
| CM CD4^+^ T cells | | 42 (33-50) | | 43 (34-52) | | | 51 (37-60) | **0.004** | 0.264 | **0.001** | **0.006** |
| EM CD4^+^ T cells | | 10 (7-14) | | 11 (7-15) | | | 9 (4-15) | 0.109 | 0.531 | 0.055 | **0.038** |
| TEMRA CD4^+^ T cells | | 1 (0-2) | | 1 (0-2) | | | 1 (0-3) | 0.683 | 0.786 | 0.382 | 0.498 |
| **T_reg_ cell subsets, % of CD4^+^ T cells** | | | |  | | |  |  |  |  |  |
| T_reg_ cells | | 3.90 (3.26-4.74) | | 3.80 (3.17-4.62) | | | 3.75 (2.98-4.61) | 0.265 | 0.184 | 0.268 | 0.584 |
| Naïve T_reg_ cells | | 1.53 (1.15-2.06) | | 1.50 (1.07-1.97) | | | 1.22 (0.78-1.72) | **0.003** | 0.122 | **0.001** | **0.017** |
| Memory T_reg_ cells | | 2.27 (1.82-2.81) | | 2.24 (1.82-2.83) | | | 2.40 (1.90-3.14) | 0.710 | 0.647 | 0.554 | 0.401 |
| **CD8^+^ T cell subsets,** **% of CD8^+^ T cells** | | | | |  |  | | | | |  |
| Naïve CD8^+^ T cells | | 17 (9-30) | | 15 (9-24) | | | 12 (7-23) | **0.008** | **0.021** | **0.019** | 0.165 |
| CM CD8^+^ T cells | | 6 (4-9) | | 6 (4-8) | | | 5 (2-8) | 0.062 | 0.132 | 0.050 | 0.153 |
| EM CD8^+^ T cells | | 41 (28-54) | | 42 (30-56) | | | 50 (31-59) | 0.228 | 0.212 | 0.190 | 0.382 |
| TEMRA CD8^+^ T cells | | 24 (13-41) | | 27 (14-42) | | | 27 (15-39) | 0.468 | 0.221 | 0.763 | 0.809 |
| **NK cells subsets,** **% of lymphocytes** | | | | |  |  | | | | |  |
| CD56^bright^ NK cells | | 0.97 (0.61-1.39) | | 0.91 (0.56-1.28) | | | 0.66 (0.54-1.02) | **0.005** | 0.058 | **0.004** | 0.051 |
| CD56^dim^ NK cells | | 9 (6-12) | | 9 (6-13) | | | 9 (7-15) | 0.291 | 0.607 | 0.133 | 0.172 |
| **CD56^+^ T cell subsets** | | |  |  | | |  |  |  |  |  |
| CD56^+^ T cells,  % of T cells | | 8 (4-13) | | 8 (4-13) | | | 7 (4-13) | 0.901 | 0.876 | 0.697 | 0.631 |
| CD4^+^ CD56^+^ T cells,  % CD4^+^ T cells | | 1 (0-2) | | 1 (0-3) | | | 1 (0-2) | 0.583 | 0.379 | 0.496 | 0.855 |
| CD8^+^ CD56^+^ T cells,  % CD8^+^ T cells | | 17 (10-25) | | 17 (11-26) | | | 17 (11-26) | 0.936 | 0.724 | 0.987 | 0.848 |

Multiple group comparisons were performed using the Kruskal-Wallis test. Two-group comparisons were performed using Mann-Whitney U test, respectively. Data is given as median (interquartile range). Statistically significant *p*-values were presented in bold. NK, natural killer; CM, central memory; EM, effector memory; TEMRA, terminally differentiated effector memory T cells re‑expressing CD45RA; T_reg_, regulatory CD4^+^ T.

**Supplementary Table 6**. **Immune cell composition in subjects stratified by coronary artery calcium score categories.**

|  | **CAC score**  **= 0 (n=627)** | **CAC score**  **= 1-10 (n=85)** | **CAC score**  **= 11-100 (n=154)** | **CAC score**  **= 101-400 (n=74)** | | | **CAC score**  **> 400 (n=27)** | *p*-value |
| --- | --- | --- | --- | --- | --- | --- | --- | --- |
| *Major cell types* | | | | | | | | |
| Leukocytes $\text{×10}^{\text{6}}\text{cells/L }$ | 5611 (4673-6637) | 5685 (4739-6419) | 5749 (4785-6584) | 5927 (5246-7010) | | | 6111 (4888-7662) | 0.098 |
| Lymphocytes $\text{ ×10}^{\text{6}}\text{cells/L}$ | 1756 (1436-2082) | 1674 (1447-2045) | 1785 (1508-2179) | 1842 (1634-2136) | | | 1881 (1559-2312) | 0.194 |
| Granulocytes $\text{×10}^{\text{6}}\text{cells/L }$ | 3214 (2538-3990) | 3148 (2659-3945) | 3280 (2568-3955) | 3444 (2813-4286) | | | 3370 (2823-4808) | 0.282 |
| Granulocyte/  Lymphocyte ratio | 1.84 (1.40-2.34) | 1.79 (1.50-2.20) | 1.79 (1.42-2.26) | 1.79 (1.49-2.28) | | | 1.88 (1.41-2.47) | 0.887 |
| Monocytes $\text{×10}^{\text{6}}\text{cells/L}$ | 361 (290-449) | 367 (313-470) | 396 (311-473) | 406 (328-482) | | | 446 (355-543) | **0.001** |
| B cells  % of lymphocytes | 11 (8-14) | 11 (8-13) | 10 (8-13) | 10 (8-13) | | | 10 (8-14) | 0.579 |
| NK cells (CD56^+^CD3^-^)  % of lymphocytes | 12 (8-16) | 12 (8-16) | 11 (8-17) | 12 (8-15) | | | 10 (8-15) | 0.947 |
| T cells  % of lymphocytes | 76 (72-81) | 78 (73-82) | 77 (72-81) | 77 (72-81) | | | 77 (73-81) | 0.825 |
| CD4^+^ T cells  % of lymphocytes | 50 (44-56) | 51 (45-57) | 50 (43-55) | 50 (47-55) | | | 48 (43-57) | 0.765 |
| CD8^+^ T cells  % of lymphocytes | 24 (19-31) | 23 (18-28) | 25 (19-32) | 24 (18-30) | | | 24 (18-35) | 0.476 |
| *Cell subsets* | | | | | | | | |
| **CD4^+^ T cell subsets.** **% of CD4^+^ T cells** | |  |  |  |  |  |  |  |
| Naïve CD4^+^ T cells | 43 (32-54) | 42 (31-56) | 42 (33-49) | 40 (30-50) | | | 36 (27-44) | 0.109 |
| CM CD4^+^ T cells | 42 (33-50) | 42 (33-53) | 43 (35-53) | 45 (35-51) | | | 49 (38-54) | 0.102 |
| EM CD4^+^ T cells | 10 (7-14) | 10 (7-16) | 11 (8-15) | 11 (6-16) | | | 10 (6-13) | 0.678 |
| TEMRA CD4^+^ T cells | 1 (0-2) | 1 (0-1) | 1 (0-2) | 2 (0-4) | | | 1 (0-2) | 0.028 |
| **T_reg_ cell subsets. % of CD4^+^ T cells** | |  |  |  | | |  |  |
| T_reg_ cells | 3.89 (3.25-4.74) | 3.81 (3.19-4.56) | 3.91 (3.27-4.73) | 3.76 (3.01-4.51) | | | 3.72 (3.29-4.76) | 0.364 |
| Naïve T_reg_ cells | 1.53 (1.14-2.04) | 1.53 (1.06-1.95) | 1.51 (1.06-2.01) | 1.31 (0.95-1.74) | | | 1.27 (1.08-1.92) | 0.104 |
| Memory T_reg_ cells | 2.27 (1.81-2.81) | 2.18 (1.83-2.85) | 2.31 (1.87-2.90) | 2.19 (1.87-2.55) | | | 2.56 (1.75-3.14) | 0.747 |
| **CD8^+^ T cell subsets. % of CD8^+^ T cells** | |  |  |  | | |  |  |
| Naïve CD8^+^ T cells | 17 (9-30) | 17 (9-24) | 16 (10-24) | 12 (7-22) | | | 12 (10-23) | **0.036** |
| CM CD8^+^ T cells | 6 (4-9) | 6 (4-9) | 6 (4-8) | 6 (3-9) | | | 6 (4-7) | 0.260 |
| EM CD8^+^ T cells | 40 (28-54) | 44 (30-58) | 43 (30-57) | 42 (29-56) | | | 46 (30-57) | 0.643 |
| TEMRA CD8^+^ T cells | 25 (13-40) | 26 (12-37) | 26 (15-43) | 28 (15-44) | | | 30 (12-50) | 0.598 |
| **NK cell subsets. % of lymphocytes** | |  |  |  |  |  |  |  |
| CD56^bright^ NK cells | 0.97 (0.61-1.39) | 0.97 (0.56-1.36) | 0.86 (0.54-1.24) | 0.86 (0.55-1.33) | | | 0.74 (0.59-1.49) | 0.116 |
| CD56^dim^ NK cells | 9 (6-12) | 9 (6-12) | 9 (6-14) | 9 (6-12) | | | 9 (6-11) | 0.786 |
| **CD56^+^ T cell subsets** | |  |  |  |  |  | | |
| CD56^+^ T cells  % of T cells | 8 (4-13) | 7 (4-12) | 8 (5-14) | 8 (4-14) | | | 8 (5-13) | 0.645 |
| CD4^+^ CD56^+^ T cells  % CD4^+^ T cells | 0 (1-2) | 1 (0-2) | 0 (1-3) | 1 (0-4) | | | 1 (0-2) | 0.418 |
| CD8^+^ CD56^+^ T cells  % CD8^+^ T cells | 17 (10-25) | 16 (9-27) | 18 (11-28) | 18 (10-24) | | | 15 (12-20) | 0.719 |

All 5 categories were compared using the Kruskal-Wallis test. Data is given as median (interquartile range). Statistically significant *p*-values were presented in bold. NK, natural killer; CM, central memory; EM, effector memory; TEMRA, terminally differentiated effector memory T cells re‑expressing CD45RA; T_reg_, regulatory CD4^+^ T.

**Supplementary Table 7. Logistic regression models between the major immune cell types and atherosclerosis burden, assessed by coronary computed tomography angiography (n=967).**

|  |  | **No stenosis vs <50% stenosis** | |  | **No stenosis vs ≥50% stenosis** | |
| --- | --- | --- | --- | --- | --- | --- |
| **Major cell types^2^** | Adjustment models | OR^1^  (95% CI) | *p*-value |  | OR^1^  (95% CI) | *p*-value |
| Leukocytes, | Model 0 | 1.061 (1.007; 1.119) | **0.027** |  | 1.115 (0.989; 1.257) | 0.076 |
| $\text{×10}^{\text{6}}\text{cells/L}$ | Model 1 | 1.029 (0.973; 1.087) | 0.315 |  | 1.053 (0.934; 1.187) | 0.402 |
|  | Model 2 | 0.990 (0.933; 1.051) | 0.748 |  | 1.008 (0.887; 1.145) | 0.906 |
|  |  |  |  |  |  |  |
| Lymphocytes, | Model 0 | 1.040 (0.992; 1.090) | 0.105 |  | 1.068 (0.958; 1.190) | 0.233 |
| $\text{×10}^{\text{6}}\text{cells/L}$ | Model 1 | 1.044 (0.994; 1.097) | 0.087 |  | 1.075 (0.964; 1.199) | 0.193 |
|  | Model 2 | 1.011 (0.959; 1.066) | 0.672 |  | 1.044 (0.929; 1.173) | 0.467 |
|  |  |  |  |  |  |  |
| Granulocytes, | Model 0 | 1.034 (0.994; 1.076) | 0.100 |  | 1.075 (0.982; 1.177) | 0.119 |
| $\text{×10}^{\text{6}}\text{cells/L}$ | Model 1 | 1.008 (0.967; 1.052) | 0.695 |  | 1.027 (0.936; 1.126) | 0.578 |
|  | Model 2 | 0.986 (0.943; 1.031) | 0.541 |  | 0.998 (0.906; 1.099) | 0.966 |
|  |  |  |  |  |  |  |
| Granulocyte/ | Model 0 | 1.004 (0.970; 1.040) | 0.811 |  | 1.020 (0.942; 1.104) | 0.633 |
| Lymphocyte ratio | Model 1 | 0.982 (0.946; 1.019) | 0.342 |  | 0.979 (0.899; 1.065) | 0.616 |
|  | Model 2 | 0.983 (0.946; 1.023) | 0.402 |  | 0.975 (0.894; 1.063) | 0.568 |
|  |  |  |  |  |  |  |
| Monocytes, | Model 0 | 1.084 (1.038; 1.131) | **<0.001** |  | 1.068 (0.969; 1.177) | 0.188 |
| $\text{×10}^{\text{6}}\text{cells/L}$ | Model 1 | 1.033 (0.986; 1.082) | 0.173 |  | 0.975 (0.880; 1.081) | 0.634 |
|  | Model 2 | 1.011 (0.963; 1.062) | 0.657 |  | 0.947 (0.851; 1.053) | 0.311 |
|  |  |  |  |  |  |  |
| B cells, | Model 0 | 0.988 (0.958; 1.020) | 0.463 |  | 0.987 (0.920; 1.060) | 0.720 |
| % of lymphocytes | Model 1 | 1.001 (0.969; 1.034) | 0.961 |  | 1.010 (0.942; 1.082) | 0.785 |
|  | Model 2 | 1.000 (0.966; 1.035) | 0.998 |  | 1.016 (0.945; 1.093) | 0.663 |
|  |  |  |  |  |  |  |
| NK cells | Model 0 | 1.001 (0.974; 1.028) | 0.964 |  | 1.060 (0.992; 1.132) | 0.084 |
| % of lymphocytes | Model 1 | 0.978 (0.950; 1.007) | 0.133 |  | 1.014 (0.949; 1.084) | 0.672 |
|  | Model 2 | 0.985 (0.955; 1.016) | 0.344 |  | 1.049 (0.976; 1.128) | 0.190 |
|  |  |  |  |  |  |  |
| T cells, | Model 0 | 1.042 (0.918; 1.183) | 0.524 |  | 0.799 (0.645; 0.990) | **0.040** |
| % of lymphocytes | Model 1 | 1.122 (0.983; 1.281) | 0.089 |  | 0.901 (0.721; 1.125) | 0.357 |
|  | Model 2 | 1.108 (0.958; 1.280) | 0.166 |  | 0.834 (0.665; 1.046) | 0.116 |
|  |  |  |  |  |  |  |
| CD4^+^ T cells, | Model 0 | 1.012 (0.945; 1.083) | 0.739 |  | 0.927 (0.804; 1.069) | 0.298 |
| % of lymphocytes | Model 1 | 1.050 (0.977; 1.129) | 0.183 |  | 0.993 (0.858; 1.150) | 0.925 |
|  | Model 2 | 1.048 (0.970; 1.131) | 0.234 |  | 0.971 (0.834; 1.130) | 0.701 |
|  |  |  |  |  |  |  |
| CD8^+^ T cells, | Model 0 | 0.995 (0.960; 1.031) | 0.764 |  | 0.943 (0.871; 1.020) | 0.142 |
| % of lymphocytes | Model 1 | 0.997 (0.960; 1.035) | 0.875 |  | 0.948 (0.876; 1.026) | 0.189 |
|  | Model 2 | 0.988 (0.947; 1.031) | 0.583 |  | 0.917 (0.840; 1.001) | 0.054 |

Model 0 – No adjustment.
Model 1 – Adjusted for age and sex.
Model 2 – Adjusted for age, sex, abdominal obesity, antihypertensive medication, smoking status, self-reported diabetes, plasma levels of high-density lipoprotein-cholesterol, plasma levels of interleukin-18, and CMV seropositivity.
^1^ Odds ratio (OR) at 10% relative increase in the cell counts or proportions.
^2^ All markers were log_10_-transformed before regression analyses.
Statistically significant *p*-values were presented in bold. CI, confidence interval; NK, natural killer; CMV, Cytomegalovirus.

**Supplementary Table 8. Logistic regression models between immune cell composition and coronary atherosclerosis burden, assessed by coronary computed tomography angiography (n=967).**

|  |  | **No stenosis vs <50% stenosis** | |  | **No stenosis vs ≥50% stenosis** | |
| --- | --- | --- | --- | --- | --- | --- |
| **Immune cell subsets^2^** | Adjustment models | OR^1^  (95% CI) | *p*-value |  | OR^1^  (95% CI) | *p*-value |
| Naïve CD4^+^ T cells, | Model 0 | 0.986 (0.955; 1.018) | 0.388 |  | 0.913 (0.856; 0.974) | **0.006** |
| % of CD4^+^ T cells | Model 1 | 1.007 (0.973; 1.042) | 0.699 |  | 0.947 (0.885; 1.013) | 0.112 |
|  | Model 2 | 1.021 (0.984; 1.059) | 0.268 |  | 0.952 (0.888; 1.021) | 0.170 |
|  |  |  |  |  |  |  |
| CM CD4^+^ T cells, | Model 0 | 1.017 (0.977; 1.058) | 0.408 |  | 1.191 (1.062; 1.335) | **0.003** |
| % of CD4^+^ T cells | Model 1 | 1.001 (0.961; 1.041) | 0.980 |  | 1.142 (1.017; 1.283) | **0.025** |
|  | Model 2 | 1.000 (0.956; 1.045) | 0.989 |  | 1.147 (1.015; 1.295) | **0.027** |
|  |  |  |  |  |  |  |
| EM CD4^+^ T cells, | Model 0 | 1.002 (0.980; 1.025) | 0.866 |  | 0.950 (0.909; 0.993) | **0.022** |
| % of CD4^+^ T cells | Model 1 | 0.998 (0.975; 1.022) | 0.865 |  | 0.950 (0.909; 0.993) | **0.023** |
|  | Model 2 | 0.995 (0.969; 1.021) | 0.693 |  | 0.934 (0.889; 0.981) | **0.007** |
|  |  |  |  |  |  |  |
| TEMRA CD4^+^ T cells, | Model 0 | 1.001 (0.992; 1.011) | 0.780 |  | 1.009 (0.986; 1.032) | 0.437 |
| % of CD4^+^ T cells | Model 1 | 1.001 (0.990; 1.011) | 0.883 |  | 1.008 (0.985; 1.030) | 0.503 |
|  | Model 2 | 0.998 (0.987; 1.010) | 0.801 |  | 1.005 (0.980; 1.031) | 0.681 |
|  |  |  |  |  |  |  |
| T_reg_ cells, | Model 0 | 0.963 (0.919; 1.010) | 0.121 |  | 0.925 (0.830; 1.030) | 0.154 |
| % of CD4^+^ T cells | Model 1 | 0.970 (0.923; 1.019) | 0.227 |  | 0.934 (0.835; 1.045) | 0.232 |
|  | Model 2 | 0.979 (0.930; 1.031) | 0.423 |  | 0.939 (0.837; 1.053) | 0.279 |
|  |  |  |  |  |  |  |
| Naïve T_reg_ cells, | Model 0 | 0.973 (0.944; 1.003) | 0.081 |  | 0.882 (0.824; 0.943) | **<0.001** |
| % of CD4^+^ T cells | Model 1 | 0.994 (0.961; 1.027) | 0.702 |  | 0.906 (0.844; 0.972) | **0.006** |
|  | Model 2 | 1.004 (0.970; 1.040) | 0.800 |  | 0.908 (0.844; 0.977) | **0.010** |
|  |  |  |  |  |  |  |
| Memory T_reg_ cells, | Model 0 | 0.982 (0.947; 1.018) | 0.326 |  | 1.012 (0.930; 1.102) | 0.775 |
| % of CD4^+^ T cells | Model 1 | 0.970 (0.933; 1.008) | 0.116 |  | 0.993 (0.912; 1.081) | 0.870 |
|  | Model 2 | 0.972 (0.933; 1.013) | 0.179 |  | 0.993 (0.907; 1.086) | 0.875 |
|  |  |  |  |  |  |  |
| Naïve CD8^+^ T cells, | Model 0 | 0.980 (0.964; 0.996) | **0.016** |  | 0.952 (0.918; 0.986) | **0.006** |
| % of CD8^+^ T cells | Model 1 | 1.000 (0.982; 1.018) | 0.986 |  | 0.981 (0.945; 1.019) | 0.322 |
|  | Model 2 | 1.010 (0.991; 1.030) | 0.300 |  | 0.991 (0.953; 1.032) | 0.672 |
|  |  |  |  |  |  |  |
| CM CD8^+^ T cells, | Model 0 | 0.993 (0.975; 1.011) | 0.447 |  | 0.950 (0.917; 0.983) | **0.003** |
| % of CD8^+^ T cells | Model 1 | 1.001 (0.982; 1.021) | 0.881 |  | 0.965 (0.931; 1.000) | **0.048** |
|  | Model 2 | 1.009 (0.989; 1.030) | 0.390 |  | 0.967 (0.931; 1.003) | 0.075 |
|  |  |  |  |  |  |  |
| EM CD8^+^ T cells, | Model 0 | 1.000 (0.979; 1.022) | 0.979 |  | 0.992 (0.949; 1.038) | 0.732 |
| % of CD8^+^ T cells | Model 1 | 0.998 (0.976; 1.020) | 0.857 |  | 0.989 (0.948; 1.032) | 0.619 |
|  | Model 2 | 0.998 (0.975; 1.021) | 0.859 |  | 0.989 (0.948; 1.033) | 0.628 |
|  |  |  |  |  |  |  |
| TEMRA CD8^+^ T cells, | Model 0 | 1.010 (0.994; 1.027) | 0.219 |  | 1.001 (0.965; 1.038) | 0.962 |
| % of CD8^+^ T cells | Model 1 | 1.003 (0.986; 1.020) | 0.747 |  | 0.993 (0.959; 1.028) | 0.699 |
|  | Model 2 | 0.997 (0.978; 1.015) | 0.722 |  | 0.986 (0.950; 1.022) | 0.438 |
|  |  |  |  |  |  |  |
| CD56^bright^ NK cells, | Model 0 | 0.976 (0.954; 0.999) | **0.043** |  | 0.927 (0.880; 0.977) | **0.005** |
| % of lymphocytes | Model 1 | 0.982 (0.958; 1.006) | 0.133 |  | 0.936 (0.887; 0.987) | **0.015** |
|  | Model 2 | 0.989 (0.964; 1.014) | 0.373 |  | 0.944 (0.893; 0.998) | **0.044** |
|  |  |  |  |  |  |  |
| CD56^dim^ NK cells, | Model 0 | 1.009 (0.986; 1.032) | 0.468 |  | 1.051 (0.993; 1.113) | 0.087 |
| % of lymphocytes | Model 1 | 0.992 (0.968; 1.016) | 0.507 |  | 1.016 (0.960; 1.076) | 0.578 |
|  | Model 2 | 0.997 (0.971; 1.023) | 0.827 |  | 1.046 (0.983; 1.113) | 0.156 |
|  |  |  |  |  |  |  |
| CD56^+^ T cells, | Model 0 | 1.003 (0.988; 1.019) | 0.674 |  | 0.990 (0.955; 1.025) | 0.565 |
| % of T cells | Model 1 | 0.999 (0.982; 1.015) | 0.861 |  | 0.981 (0.947; 1.017) | 0.297 |
|  | Model 2 | 0.997 (0.978; 1.016) | 0.720 |  | 0.970 (0.931; 1.010) | 0.143 |
|  |  |  |  |  |  |  |
| CD4^+^ CD56^+^ T cells, | Model 0 | 1.003 (0.994; 1.012) | 0.519 |  | 1.004 (0.984; 1.025) | 0.687 |
| % of CD4^+^ T cells | Model 1 | 1.003 (0.993; 1.012) | 0.597 |  | 1.004 (0.983; 1.025) | 0.729 |
|  | Model 2 | 1.001 (0.989; 1.012) | 0.891 |  | 1.000 (0.976; 1.025) | 0.978 |
|  |  |  |  |  |  |  |
| CD8^+^ CD56^+^ T cells, | Model 0 | 1.005 (0.986; 1.024) | 0.628 |  | 0.991 (0.950; 1.035) | 0.683 |
| % of CD8^+^ T cells | Model 1 | 0.995 (0.975; 1.016) | 0.650 |  | 0.976 (0.934; 1.019) | 0.263 |
|  | Model 2 | 0.991 (0.969; 1.013) | 0.411 |  | 0.962 (0.918; 1.010) | 0.117 |

Model 0 – No adjustment.
Model 1 – Adjusted for age and sex.
Model 2 – Adjusted for age, sex, abdominal obesity, antihypertensive medication, smoking status, self-reported diabetes, plasma levels of high-density lipoprotein-cholesterol, plasma levels of interleukin-18, and CMV seropositivity.
^1^ Odds ratio (OR) at 10% relative increase in the cell counts and proportions.
^2^ All markers were log_10_-transformed before regression analyses.

Statistically significant *p*-values were presented in bold. CI, confidence interval; CM, central memory; EM, effector memory; TEMRA, terminally differentiated effector memory T cells re‑expressing CD45RA; T_reg_, regulatory CD4^+^ T; NK, natural killer; CMV, Cytomegalovirus.

**Supplementary Table 9. Adjustment of results using the Benjamini-Hochberg procedure**

| Model 1 | | | |  | Model 2 | | | |
| --- | --- | --- | --- | --- | --- | --- | --- | --- |
| Variable | *P* value | Rank (*i*) | BH critical values |  | Variable | *P* value | Rank (*i*) | BH critical values |
| Naïve T_reg_ cells | 0.006 | 1 | 0.010 |  | EM CD4^+^ T cells | 0.007 | 1 | 0.010 |
| CD56^bright^ NK cells | 0.015 | 2 | 0.019 |  | Naïve T_reg_ cells | 0.010 | 2 | 0.019 |
| EM CD4^+^ T cells | 0.023 | 3 | 0.029 |  | CM CD4^+^ T cells | 0.027 | 3 | 0.029 |
| CM CD4^+^ T cells | 0.025 | 4 | 0.038 |  | CD56^bright^ NK cells | 0.044 | 4 | 0.038 |
| CM CD8^+^ T cells | 0.048 | 5 | 0.048 |  | CD8^+^ T cells | 0.054 | 5 | 0.048 |
| Naïve CD4^+^ T cells | 0.112 | 6 | 0.058 |  | CM CD8^+^ T cells | 0.075 | 6 | 0.058 |
| CD8^+^ T cells | 0.189 | 7 | 0.067 |  | T cells | 0.116 | 7 | 0.067 |
| Lymphocytes | 0.193 | 8 | 0.077 |  | CD8^+^ CD56^+^ T cells | 0.117 | 8 | 0.077 |
| T_reg_ cells | 0.232 | 9 | 0.087 |  | CD56^+^ T cells | 0.143 | 9 | 0.087 |
| CD8^+^ CD56^+^ T cells | 0.263 | 10 | 0.096 |  | CD56^dim^ NK | 0.156 | 10 | 0.096 |
| CD56^+^ T cells | 0.297 | 11 | 0.106 |  | Naïve CD4^+^ T cells | 0.170 | 11 | 0.106 |
| Naïve CD8^+^ T cells | 0.322 | 12 | 0.115 |  | NK cells | 0.190 | 12 | 0.115 |
| T cells | 0.357 | 13 | 0.125 |  | T_reg_ cells | 0.279 | 13 | 0.125 |
| Leukocytes | 0.402 | 14 | 0.135 |  | Monocytes | 0.311 | 14 | 0.135 |
| TEMRA CD4^+^ T cells | 0.503 | 15 | 0.144 |  | TEMRA CD8^+^ T cells | 0.438 | 15 | 0.144 |
| Granylocytes | 0.578 | 16 | 0.154 |  | Lymphocytes | 0.467 | 16 | 0.154 |
| CD56^dim^ NK cells | 0.578 | 17 | 0.163 |  | GLR | 0.568 | 17 | 0.163 |
| GLR | 0.616 | 18 | 0.173 |  | EM CD8^+^ T cells | 0.628 | 18 | 0.173 |
| EM CD8^+^ T cells | 0.619 | 19 | 0.183 |  | B cells | 0.663 | 19 | 0.183 |
| Monocytes | 0.634 | 20 | 0.192 |  | Naïve CD8^+^ T cells | 0.672 | 20 | 0.192 |
| NK cells | 0.672 | 21 | 0.202 |  | TEMRA CD4^+^ T cells | 0.681 | 21 | 0.202 |
| TEMRA CD8^+^ T cells | 0.699 | 22 | 0.212 |  | CD4^+^ T cells | 0.701 | 22 | 0.212 |
| CD4^+^ CD56^+^ T cells | 0.729 | 23 | 0.221 |  | Memory T_reg_ cells | 0.875 | 23 | 0.221 |
| B cells | 0.785 | 24 | 0.231 |  | Leukocytes | 0.906 | 24 | 0.231 |
| Memory T_reg_ cells | 0.870 | 25 | 0.240 |  | Granylocytes | 0.966 | 25 | 0.240 |
| CD4^+^ T cells | 0.925 | 26 | 0.250 |  | CD4^+^ CD56^+^ T cells | 0.978 | 26 | 0.250 |

*p*-values generated from Model 1 and 2 of the multinomial logistic regression analyses between subjects with no stenosis and stenosis ≥ 50%. False discovery rate was 25%. Statistically significant *p*-values after adjustment for Type 1 error were highlighted in green. CM, central memory; EM, effector memory; TEMRA, terminally differentiated effector memory T cells re‑expressing CD45RA; T_reg_, regulatory CD4^+^ T; NK, natural killer; BH, Benjamini-Hochberg.

**Supplementary Table 10. Logistic regression models between immune cell composition and coronary artery calcium score (CAC) (n=967).**

|  |  | **CAC = 0 vs CAC = 101-400** | |  | **CAC = 0 vs CAC > 400** | |
| --- | --- | --- | --- | --- | --- | --- |
| **Immune cell types^2^** | Adjustment model | OR^1^  (95% CI) | *p*-value | | OR^1^  (95% CI) | *p*-value |
| Leukocytes | Model 0 | 1.090 (0.995; 1.194) | 0.065 | | 1.203 (1.040; 1.390) | **0.013** |
| $\text{×10}^{\text{6}}\text{cells/L}$ | Model 1 | 1.041 (0.949; 1.142) | 0.392 | | 1.114 (0.964; 1.288) | 0.142 |
|  |  |  |  | |  |  |
| Monocytes | Model 0 | 1.078 (1.001; 1.161) | **0.046** | | 1.219 (1.084; 1.370) | **<0.001** |
| $\text{×10}^{\text{6}}\text{cells/L}$ | Model 1 | 1.000 (0.925; 1.082) | 0.992 | | 1.098 (0.971; 1.242) | 0.137 |
|  |  |  |  | |  |  |
| Naïve CD8^+^ T cells | Model 0 | 0.963 (0.937; 0.990) | **0.007** | | 0.972 (0.930; 1.016) | 0.209 |
| % of CD8^+^ T cells | Model 1 | 0.991 (0.962; 1.020) | 0.526 | | 1.012 (0.966; 1.060) | 0.617 |

Model 0 – No adjustment.
Model 1 – Adjusted for age and gender.

^1^ Odds ratio (OR) at 10% relative increase in the cell counts or proportions.
^2^ All markers were log_10_-transformed before regression analyses.
Statistically significant *p*-values were presented in bold. CI, confidence interval; CMV, Cytomegalovirus.
